# Use of guideline‐recommended medical therapy in patients with heart failure and chronic kidney disease: from physician's prescriptions to patient's dispensations, medication adherence and persistence

**DOI:** 10.1002/ejhf.2620

**Published:** 2022-08-02

**Authors:** Roemer J. Janse, Edouard L. Fu, Ulf Dahlström, Lina Benson, Bengt Lindholm, Merel van Diepen, Friedo W. Dekker, Lars H. Lund, Juan‐Jesus Carrero, Gianluigi Savarese

**Affiliations:** ^1^ Department of Medical Epidemiology and Biostatistics Karolinska Institutet Stockholm Sweden; ^2^ Department of Clinical Epidemiology Leiden University Medical Center Leiden The Netherlands; ^3^ Division of Pharmacoepidemiology and Pharmacoeconomics Brigham and Women's Hospital and Harvard Medical School Boston MA USA; ^4^ Department of Cardiology and the Department of Health, Medicine and Caring Sciences Linkoping University Linkoping Sweden; ^5^ Division of Cardiology, Department of Medicine Karolinska Institutet Stockholm Sweden; ^6^ Renal Medicine and Baxter Novum, Department of Clinical Science, Intervention and Technology Karolinska Institutet Stockholm Sweden; ^7^ Heart, Vascular and Neuro Theme Karolinska University Hospital Stockholm Sweden; ^8^ Division of Nephrology, Department of Clinical Sciences, Karolinska Institutet Danderyd Hospital Stockholm Sweden

**Keywords:** Heart failure, Chronic kidney disease, Renin–angiotensin–aldosterone‐system inhibitors, Angiotensin receptor–neprilysin inhibitors, Beta‐blockers, Mineralocorticoid receptor antagonists

## Abstract

**Aim:**

Half of heart failure (HF) patients have chronic kidney disease (CKD) complicating their pharmacological management. We evaluated physicians' and patients' patterns of use of evidence‐based medical therapies in HF across CKD stages.

**Methods and results:**

We studied HF patients with reduced (HFrEF) and mildly reduced (HFmrEF) ejection fraction enrolled in the Swedish Heart Failure Registry in 2009–2018. We investigated the likelihood of physicians to prescribe guideline‐recommended therapies to patients with CKD, and of patients to fill the prescriptions within 90 days of incident HF (initiating therapy), to adhere (proportion of days covered ≥80%) and persist (continued use) on these treatments during the first year of therapy. We identified 31 668 patients with HFrEF (median age 74 years, 46% CKD). The proportions receiving a prescription for angiotensin‐converting enzyme inhibitors/angiotensin receptor blockers/angiotensin receptor–neprilysin inhibitors (ACEi/ARB/ARNi) were 96%, 92%, 86%, and 68%, for estimated glomerular filtration rate (eGFR) ≥60, 45–59, 30–44, and <30 ml/min/1.73 m^2^, respectively; for beta‐blockers 94%, 93%, 92%, and 92%, for mineralocorticoid receptor antagonists (MRAs) 45%, 44%, 37%, 24%; and for triple therapy (combination of ACEi/ARB/ARNi + beta‐blockers + MRA) 38%, 35%, 28%, and 15%. Patients with CKD were less likely to initiate these medications, and less likely to adhere to and persist on ACEi/ARB/ARNi, MRA, and triple therapy. Among stoppers, CKD patients were less likely to restart these medications. Results were consistent after multivariable adjustment and in patients with HFmrEF (*n* = 15 114).

**Conclusions:**

Patients with HF and CKD are less likely to be prescribed and to fill prescriptions for evidence‐based therapies, showing lower adherence and persistence, even at eGFR categories where these therapies are recommended and have shown efficacy in clinical trials.

## Graphical abstract

Patients with heart failure and chronic kidney disease are less likely to receive and persist on guideline‐recommended medical therapies.

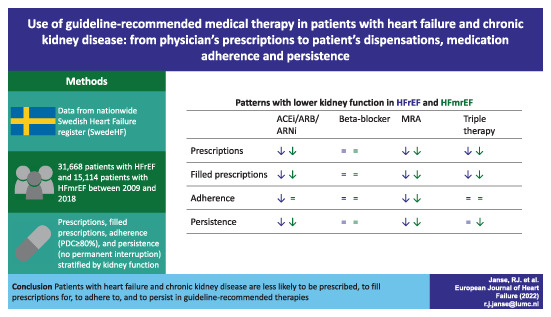

## Background

Chronic kidney disease (CKD) is a common comorbidity among patients with heart failure (HF): approximately 60% of patients with HF have an estimated glomerular filtration rate (eGFR) <60 ml/min/1.73 m^2^ ^1^ and 11% have an eGFR <30 ml/min/1.73 m^2^.^2^ The presence of CKD conveys higher risk of adverse events,^3^, ^4^ and may complicate HF pharmacological management, given that low kidney function influences drug elimination and metabolism, increasing the risk of adverse effects (e.g. the risk of hyperkalaemia).^5^

To reduce mortality/morbidity in HF, the 2021 European Society of Cardiology (ESC) guidelines on HF provide a class I recommendation for the use of renin–angiotensin–aldosterone system inhibitors (RAASi) and angiotensin receptor–neprilysin inhibitors (ARNi), beta‐blockers, and sodium–glucose cotransporter 2 inhibitors (SGLT2i) in HF with reduced ejection fraction (HFrEF), and a IIb recommendation for RAASi/ARNi and beta‐blockers in HF with mildly reduced ejection fraction (HFmrEF).^6^ While guidelines suggest caution in the use of these medications for patients with advanced CKD (eGFR <30 ml/min/1.73 m^2^), they make no distinction for milder stages of CKD severity (eGFR 30–60 ml/min/1.73 m^2^). However, therapeutic nihilism, underrepresentation of patients with CKD in clinical trials and fear of adverse effects may have an impact on the provision of guideline‐recommended treatment of patients with concomitant HF and CKD.^5^

Historical data from North America^7^, ^8^, ^9^ and Sweden^2^ have suggested that evidence‐based HF therapies are less likely to be prescribed to patients who also have CKD. More recently, Patel *et al*.^1^ evaluated practices in over 400 sites in the USA between 2014 and 2019 confirming a persistent low rate of prescription of key medications in these patients but did not investigate other dimensions of pharmacological treatment. Extrapolation of these observations to geographically distinct healthcare systems may increase generalizability and motivate strategies to improve care in this setting.

Physician's prescriptions are just one aspect of pharmacological management, and there are other factors which might limit the effectiveness of therapies. These include the patient filling the prescription (i.e. picking up a new medication at the pharmacy), taking the medication according to the prescription (i.e. adherence), and getting a new supply of the medication when the previous supply runs out (re‐filling prescriptions, i.e. persistence). We are not aware of any prior studies that evaluated these aspects of therapy management in patients with HF and CKD. Therefore, this study aims to provide a holistic assessment of the use of guideline‐recommended medical therapies in Swedish patients with HFrEF and HFmrEF across differing severity of CKD.

## Methods

### Data sources

We used data from the Swedish Heart Failure Registry (SwedeHF), an ongoing national quality register that started in 2000 and that includes both in‐ and outpatients with HF, regardless of left ventricular ejection fraction (LVEF).^10^ Until 2017, HF was defined as clinician‐judged. Thereafter, patients were enrolled according to the International Classification of Diseases, Tenth Revision (ICD‐10) codes I50.0, I50.1, I50.9, I42.0, I42.6, I42.7, I25.5, I11.0, I13.0, and I13.2.^10^ Informed consent is not required, although patients are informed of entry into Swedish quality registers and can opt out. Through linkage with the Swedish Population Register, Prescribed Drug Register, National Patient Register, and socioeconomic (LISA) database, additional information on comorbidities, medications dispensed at Swedish pharmacies, vital status, and socioeconomic characteristics was obtained. Linkage was performed by using the Swedish personal identity number that all residents, regardless of citizenship, have in Sweden. This study was approved by the Swedish Ethical Review Authority.

### Study design

We included all patients with an entry in SwedeHF during 2009–2018 to evaluate recent clinical practice in relation to the ESC guidelines from 2008 and onwards.^11^, ^12^, ^13^ Since our study focused on patients with CKD and HF, we excluded those with missing eGFR or LVEF. HFrEF was defined as LVEF <40% and HFmrEF as LVEF 40–49%, and when more than one registration per patient was available, we selected the most recent entry. Patients with HF with preserved ejection fraction (HFpEF) were excluded as there are no clear recommendations for the investigated drugs for this patient group. Finally, we excluded those who died within 90 days post register entry because death early after baseline precludes the possibility to fill prescriptions and initiate treatment.

### Study exposure

The study exposure was the patient's baseline eGFR, calculated using the 2009 CKD Epidemiology Collaboration equation^14^ from serum or plasma creatinine measurements reported at SwedeHF registration. This creatinine measurement was a creatinine test performed 24 h after discharge for inpatients, or the closest outpatient creatinine test prior to SwedeHF registration. Creatinine tests were performed using methods traceable to isotope dilution mass spectrometry standards and eGFR was stratified into four categories according to Kidney Disease: Improving Global Outcomes (KDIGO) classification^15^: normal or mildly decreased kidney function (eGFR ≥60 ml/min/1.73 m^2^), mildly to moderately decreased (eGFR 45–59 ml/min/1.73 m^2^), moderately to severely decreased (eGFR 30–44 ml/min/1.73 m^2^), and severely decreased (eGFR <30 ml/min/1.73 m^2^, including patients on maintenance dialysis).

### Study outcomes

We evaluated patterns of use of guideline‐recommended medications according to the ESC guidelines of 2008, 2012, and 2016 (online supplementary *Table* *S1*). These included: (i) angiotensin‐converting enzyme inhibitors (ACEis) or angiotensin receptor blockers (ARBs) or ARNi; (ii) beta‐blockers; (iii) mineralocorticoid receptor antagonists (MRAs). Triple therapy was defined as the combination of ACEi/ARB/ARNi, beta‐blockers, and MRAs. We performed separate analyses for ARNi use in HFrEF during the years 2016–2018 as the guideline recommendation was issued in 2016. We did not evaluate the use of SGLT2i as the indication of SGLT2i for HF had not yet been approved in Sweden during the data collection period.

For two study outcomes the date of the SwedeHF registration was the index date: *prescription*, defined as the physician's intent to treat a patient with a certain drug and recorded in the SwedeHF case registration form; *treatment initiation/filling the prescription* (i.e. compliance with the prescription by picking it up at the pharmacy), defined by a record of a pharmacy dispense in the Swedish prescribed drug register,^16^ which has complete coverage of all dispensed prescriptions at Swedish pharmacies. Some patients had not used the medications of interest prior to the SwedeHF registration. For these patients, treatment initiation was defined as a filled prescription within 90 days of the SwedeHF registration. For prevalent medication users we estimated the expected date for a new pharmacy fill based on the pill supply from the last dispensation prior to SwedeHF registration, and treatment initiation was defined as a filled prescription within 60 days after the end of the estimated pill supply.

For two additional study outcomes we used the date of the first prescription fill after the SwedeHF registration as index date: *treatment adherence* and *persistence* during the first 12 months of therapy (or until censoring). For each medication of interest, we determined adherence by assessing the proportion of days covered (PDC), a method that considers the number of consecutive fills and the distance (in days) between them. The expected duration of the fill (i.e. the number of days that one package of medication would last) was estimated through the average frequency of dispensing for each single formulation in the study population. Low adherence was defined as a PDC <80%. Non‐persistence (i.e. discontinuation of therapy) was defined by the absence of a new dispense during at least 60 days after the end of the last estimated pill supply.

Finally, among discontinuers, we evaluated the percentage of patients that *restarted* their medications within 3 months. For restarting, the date of therapy discontinuation was the index date.

### Covariates

Covariates included not only demographic and clinical characteristics commonly available in administrative registers, but also quantitative vital, laboratory and HF specific parameters. These were age, sex, smoking, socioeconomic characteristics (civil status, income, and highest attained education), period (2009–2013 or 2014–2018), New York Heart Association (NYHA) class, HF duration (<6 or >6 months), N‐terminal pro‐B‐type natriuretic peptide, hospitalization at register entry, comorbidities (obesity, anaemia, atrial fibrillation, cerebrovascular disease, chronic obstructive pulmonary disease, dilated cardiomyopathy, diabetes mellitus, hypertension, ischaemic heart disease, liver disease, peripheral artery disease, valvular disease, and cancer), procedures (coronary revascularization and devices), other ongoing medications (digoxin, diuretics, statins, anticoagulants, antiplatelets, and nitrates), and various clinical measures (body mass index, blood pressure, mean arterial pressure, haemoglobin, potassium, and heart rate). The definitions of these covariates are detailed in online supplementary *Table* *S2*.

### Statistical analysis

Continuous variables are presented as either mean (standard deviation) or median (interquartile range [IQR]), depending on the distribution. Categorical variables are presented as number (percentage). We portrayed the association of baseline eGFR categories with study outcomes using multivariable adjusted odds ratios calculated by logistic regression. The group with eGFR ≥60 ml/min/1.73 m^2^ represented the reference in our analyses. Multivariable models were adjusted for all variables with <10% missing observations. In case of missingness, categorical variables were given a missing indicator and continuous variables were given the median value of the cohort. Covariates with >10% of missingness were few and not considered in the modelling (*Table* *1*). Absolute risks were computed for persistence, taking into account the competing risk of death or migration using the Aalen–Johansen estimator.^17^

**Table 1:** Baseline characteristics for patients with heart failure with reduced ejection fraction, overall and by estimated glomerular filtration rate (in ml/min/1.73 m^2^) categories

	% of missing	Overall	eGFR ≥60	eGFR 45–59	eGFR 30–44	eGFR <30
Patients		31 668 (100)	17 032 (54)	6967 (22)	5363 (17)	2306 (7)
Age, years		74 [65–81]	69 [60–76]	77 [71–83]	80 [74–85]	80 [73–85]
Age group, years^a^						
<45		718 (2)	650 (4)	38 (1)	12 (0)	18 (1)
46–65		7500 (24)	6120 (36)	832 (12)	357 (7)	191 (8)
66–75		9386 (30)	5593 (33)	2064 (30)	1214 (23)	515 (22)
>75		14 064 (44)	4669 (27)	4033 (58)	3780 (70)	1582 (69)
Women		9052 (29)	4243 (25)	2178 (31)	1784 (33)	847 (37)
eGFR, ml/min/1.73 m^2^ ^a^		62 [46–81]	80 [69–90]	53 [49–56]	38 [35–42]	24 [19–27]
Smoking^a^	20.4%					
Never		10 562 (42)	5293 (39)	2527 (46)	1934 (46)	808 (47)
Former		11 339 (45)	6134 (45)	2448 (44)	1968 (47)	789 (46)
Current		3305 (13)	2315 (17)	555 (10)	307 (7)	128 (7)
Index year 2014–2018		18 121 (57)	9902 (58)	4044 (58)	2992 (56)	1183 (51)
Other heart failure characteristics						
NYHA class III/IV^a^	22.9%	10 251 (42)	4444 (33)	2459 (46)	2301 (57)	1047 (67)
HF duration >6 months	2.3%	17 459 (56)	7766 (47)	4175 (61)	3814 (72)	1704 (76)
NT‐proBNP, pg/ml^a^	40.5%	2490 [1026–5788]	1692 [706–3800]	2924 [1374–6189]	4350 [2065–8800]	8605 [3634–18 800]
Hospitalized at registry entry		10 072 (32)	4554 (27)	2266 (33)	2111 (39)	1141 (49)
Comorbidities						
Obesity^a^	41.9%	4346 (24)	2535 (25)	863 (22)	639 (21)	309 (23)
Atrial fibrillation		17 303 (55)	8277 (49)	4177 (60)	3443 (64)	1406 (61)
Anaemia	6.3%	9516 (32)	3643 (23)	2170 (33)	2287 (45)	1416 (64)
Cerebrovascular disease		5077 (16)	2182 (13)	1264 (18)	1133 (21)	498 (22)
COPD		4033 (13)	1974 (12)	926 (13)	797 (15)	336 (15)
Dilated cardiomyopathy		6756 (21)	4173 (25)	1329 (19)	904 (17)	350 (15)
Diabetes mellitus		8765 (28)	3936 (23)	2043 (29)	1870 (35)	916 (40)
Hypertension		20 019 (63)	9570 (56)	4701 (67)	3890 (73)	1858 (81)
Ischaemic heart disease		18 158 (57)	8483 (50)	4305 (62)	3696 (69)	1674 (73)
Liver disease	1.8%	4907 (16)	2483 (15)	1094 (16)	933 (18)	397 (18)
Peripheral artery disease		2907 (9)	1199 (7)	683 (10)	653 (12)	372 (16)
Valvular disease		7758 (24)	3412 (20)	1921 (28)	1675 (31)	750 (33)
Cancer		3761 (12)	1686 (10)	930 (13)	763 (14)	382 (17)
Procedures						
Coronary revascularization		11 566 (37)	5540 (33)	2731 (39)	2259 (42)	1036 (45)
Devices (CRT, ICD, or pacemaker)	0.9%	6867 (22)	3042 (18)	1691 (25)	1517 (29)	617 (27)
Medication						
Digoxin	0.4%	4343 (14)	2530 (15)	985 (14)	664 (12)	164 (7)
Diuretics	0.5%	24 194 (77)	11 387 (67)	5767 (83)	4897 (92)	2143 (94)
Statins	0.3%	16 489 (52)	8633 (51)	3746 (54)	2914 (54)	1196 (52)
Anticoagulants	0.4%	15 297 (48)	7779 (46)	3647 (53)	2842 (53)	1029 (45)
Antiplatelets	0.5%	13 153 (42)	7031 (41)	2860 (41)	2180 (41)	1082 (47)
Nitrates	0.4%	3638 (12)	1224 (7)	938 (14)	936 (18)	540 (24)
Clinical measures						
BMI, kg/m^2^ ^a^	41.9%	26 [23–30]	26 [23–30]	26 [23–29]	26 [23–29]	26 [23–30]
Systolic BP, mmHg	1.6%	120 [110–136]	120 [110–138]	120 [110–136]	120 [110–134]	120 [110–138]
Diastolic BP, mmHg	1.5%	70 [65–80]	74 [65–80]	70 [62–80]	70 [60–80]	70 [60–80]
Mean arterial pressure, mmHg^a^	1.5%	90 (13)	91 (13)	89 (13)	87 (13)	88 (13)
Haemoglobin, g/L	6.3%	134 (17)	138 (17)	133 (16)	128 (17)	121 (17)
Potassium, mmol/L^a^	10.5%	4 (0)	4 (0)	4 (0)	4 (0)	4 (1)
Heart rate, bpm	2.5%	71 [62–81]	70 [62–81]	71 [62–81]	71 [63–80]	72 [64–81]
Socioeconomic characteristics						
Single civil status	0.2%	14 613 (46)	7605 (45)	3244 (47)	2609 (49)	1155 (50)
Income above median		18 600 (59)	10 805 (63)	3869 (56)	2781 (52)	1145 (50)
Highest attained education	1.8%					
Compulsory school		12 968 (42)	6219 (37)	3060 (45)	2588 (49)	1101 (49)
Secondary school		12 794 (41)	7423 (44)	2642 (39)	1913 (36)	816 (36)
University		5324 (17)	3107 (19)	1136 (17)	758 (14)	323 (14)

Categorical variables are presented as percentage. Continuous variables are presented as mean (standard deviation) or median [interquartile range], depending on the distribution.

BMI, body mass index; BP, blood pressure; COPD, chronic obstructive pulmonary disease; CRT, cardiac resynchronization therapy; eGFR, estimated glomerular filtration rate; ICD, implantable cardioverter‐defibrillator; IQR, interquartile range; NT‐proBNP, N‐terminal pro‐B‐type natriuretic peptide; NYHA, New York Heart Association; SD, standard deviation.

^a^ Not adjusted for in analyses.

As a sensitivity analysis, we explored the consistency of our findings through evaluating separately the distinct periods where ESC guideline recommendations were issued (i.e. 2009–2011, 2012–2015, and 2016–2018). As a supporting analysis, we evaluated whether suboptimal management of HF patients with CKD expanded to other comorbid conditions. Specifically, we explored the prescription of anticoagulation medication among patients with atrial fibrillation (definitions in online supplementary *Table* *S2*). All statistical analyses were performed using R version 4.1.2 (R Foundation for Statistical Computing, Vienna, Austria).

## Results

After applying inclusion and exclusion criteria, we included 31 668 patients with HFrEF and 15 114 with HFmrEF (online supplementary *Figure* *S1*). The median age (IQR) was 74 (65–81) and 76 (67–83) years and 29% and 37% of participants were female for the HFrEF and HFmrEF cohorts, respectively (*Table* *1* and online supplementary *Table* *S3*). In both groups, the most common comorbidities were hypertension (63% in HFrEF and 70% in HFmrEF), ischaemic heart disease (57% and 56%), and atrial fibrillation (55% and 59%). A total of 46% of patients had CKD (eGFR <60 ml/min/1.73 m^2^) in both HFrEF and HFmrEF. Commonly prescribed drugs were diuretics (77% and 70%) and statins (52% and 52%). From 2011 onwards, data on diuretics subclasses were available. In HFrEF patients included after 2011, of 76.1% patients prescribed diuretics, 98.4% were prescribed loop diuretics. In HFmrEF, of 69.2% of patients included after 2011 prescribed diuretics, 97% were on loop diuretics.

### Physician prescriptions

**Figure 1:**
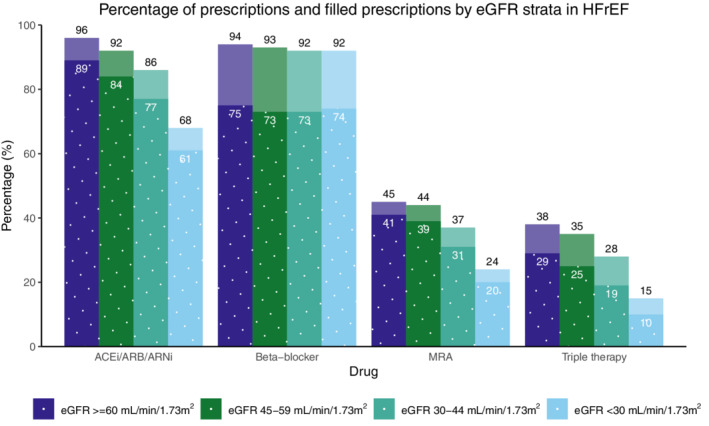
Percentages of issued prescriptions at register entry (light bars) and filled prescriptions (i.e. initiation of treatments) within 90 days after index date (dotted dark bars) by estimated glomerular filtration rate (eGFR) strata among patients with heart failure with reduced ejection fraction (HFrEF). ACEi, angiotensin‐converting enzyme inhibitor; ARB, angiotensin receptor blocker; ARNi, angiotensin receptor–neprilysin inhibitor; MRA, mineralocorticoid receptor antagonist.

**Figure 2:**
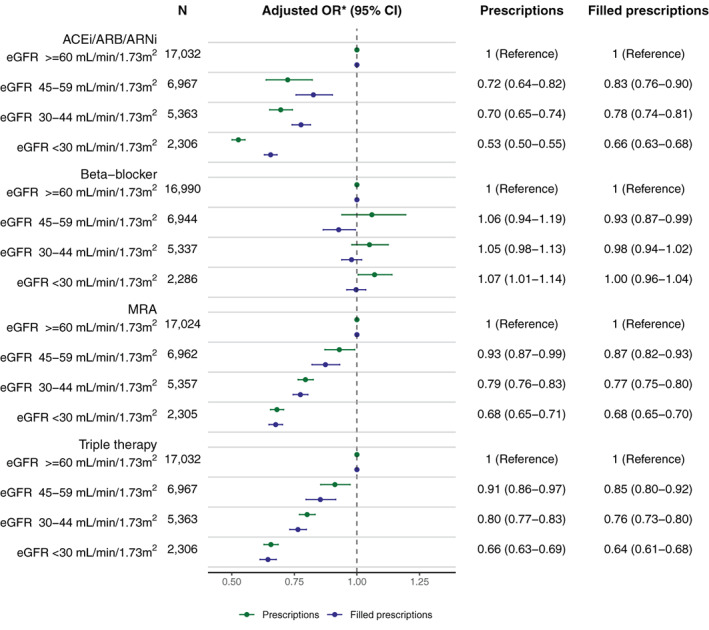
Multivariable adjusted odds ratios (OR) (and 95% confidence intervals [CI]) for being prescribed and filling the prescription of guideline‐recommended therapies in patients with heart failure with reduced ejection fraction and differing estimated glomerular filtration rate (eGFR) categories. ACEi, angiotensin‐converting enzyme inhibitor; ARB, angiotensin receptor blocker; ARNi, angiotensin receptor–neprilysin inhibitor; MRA, mineralocorticoid receptor antagonist. *Adjusted for age, sex, hospitalization at index, heart failure duration, anaemia, atrial fibrillation, cerebrovascular disease, chronic obstructive pulmonary disease, diabetes mellitus, dilated cardiomyopathy, hypertension, ischaemic heart disease, liver disease, peripheral artery disease, valvular disease, cancer, coronary revascularization, devices (cardiac resynchronization therapy, implantable cardioverter‐defibrillator, or pacemaker), prescription for digoxin, diuretics, statins, anticoagulants, antiplatelets, or nitrates at index, heart rate, systolic and diastolic blood pressure, haemoglobin, highest achieved education, civil status, income, and year of index category.

The percentage of patients prescribed (either initiated for incident users or continued for prevalent users) with ACEi/ARB/ARNi and MRAs but not for beta‐blockers decreased with lower kidney function. In HFrEF, the proportions prescribed ACEi/ARB/ARNi were 96%, 92%, 86%, and 68% for eGFR ≥60, 45–59, 30–44, and < 30 ml/min/1.73 m^2^, respectively (*Figure* *1*). For beta‐blockers this was 94%, 93%, 92%, and 92%; for MRAs 45%, 44%, 37%, and 24%; and for triple therapy 38%, 35%, 28%, and 15%. Compared to patients with normal kidney function (eGFR ≥60 ml/min/1.73 m^2^) these differences persisted after adjusting for comorbidities and medication use in multivariable analyses for all CKD stages (*Figure* *2*). Similar trends were observed in patients with HFmrEF (online supplementary *Figures* *S2* and *S3*).

### Initiation of treatments

Patients with CKD were less likely to fill their prescriptions for ACEi/ARB/ARNi and MRAs but not for beta‐blockers. Specifically, prescriptions of ACEi/ARB/ARNi were filled by 89%, 84%, 77%, and 61% of patients for eGFR ≥60, 45–59, 30–44, and <30 ml/min/1.73 m^2^, respectively; 75%, 73%, 73%, and 74%; for beta‐blockers; 41%, 39%, 31%, and 20% for MRAs; and 29%, 25%, 19%, and 10% for triple therapy. Compared to patients with normal kidney function (eGFR ≥60 ml/min/1.73 m^2^) these differences persisted after adjusting for comorbidities and medication use in multivariable analyses for all CKD stages (*Figure* *2*). Similar trends were observed in patients with HFmrEF (online supplementary *Figures* *S2* and *S3*).

### Adherence to treatment

**Figure 3:**
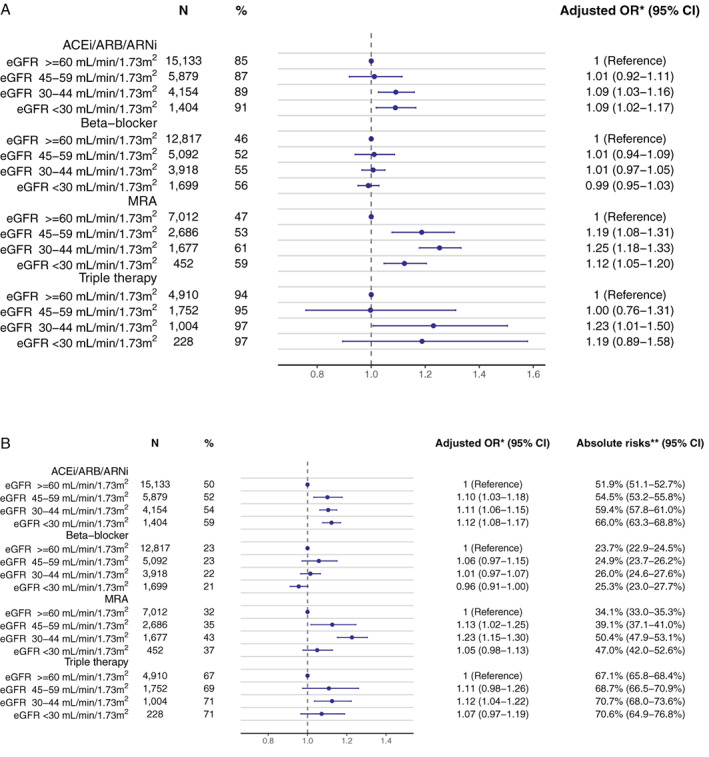
(*A*) Multivariable adjusted odds ratios (OR) (and 95% confidence intervals [CI]) for low adherence (proportion of days covered <80%) to guideline‐recommended therapies during the first year of therapy in patients with heart failure with reduced ejection fraction and differing categories of estimated glomerular filtration rate (eGFR). (*B*) Absolute risks and multivariable adjusted OR (with 95% CI) for non‐persistence (i.e. treatment discontinuation) to guideline‐recommended therapies during the first year of therapy in patients with heart failure with reduced ejection fraction and differing eGFR categories. ACEi, angiotensin‐converting enzyme inhibitor; ARB, angiotensin receptor blocker; ARNi, angiotensin receptor–neprilysin inhibitor; MRA, mineralocorticoid receptor antagonist. *Adjusted for age, sex, hospitalization at index, heart failure duration, anaemia, atrial fibrillation, cerebrovascular disease, chronic obstructive pulmonary disease, diabetes mellitus, dilated cardiomyopathy, hypertension, ischaemic heart disease, liver disease, peripheral artery disease, valvular disease, cancer, coronary revascularization, devices (cardiac resynchronization therapy, implantable cardioverter‐defibrillator, or pacemaker), prescription for digoxin, diuretics, statins, anticoagulants, antiplatelets, or nitrates at index, heart rate, systolic and diastolic blood pressure, haemoglobin, highest achieved education, civil status, income, and year of index category. **Takes into account censoring and the competing risk of death.

Patients with CKD were more likely to have low adherence (i.e. PDC <80%) to guideline‐recommended therapies. For ACEi/ARB/ARNi, 85%, 87%, 89%, and 91% of patients showed low adherence for eGFR ≥60, 45–59, 30–44, and <30 ml/min/1.73 m^2^, respectively (*Figure* *3*). For beta‐blockers this was 46%, 52%, 55%, and 56%; for MRAs 47%, 53%, 61%, and 59%; and for triple therapy 94%, 95%, 97%, and 97%. These differences remained after multivariable analysis for ACEi/ARB/ARNi and MRAs, while no differences were observed for beta‐blockers, and confidence intervals were broad for triple therapy (*Figure* *3A*). Results were similar for patients with HFmrEF (online supplementary *Figure* *S4A*).

### Discontinuation of treatments

Patients with CKD were more likely to discontinue ACEi/ARB/ARNi and MRA but not beta‐blockers. In HFrEF, for ACEi/ARB/ARNi, 50%, 52%, 54%, and 59% of patients had low persistence (i.e. discontinued) for eGFR ≥60, 45–59, 30–44, and <30 ml/min/1.73 m^2^, respectively (*Figure* *3B*). For beta‐blockers, this was 23%, 23%, 22%, and 21%; for MRAs 32%, 35%, 43%, and 37%; and for triple therapy 67%, 69%, 71%, and 71%. These results were consistent after multivariable adjustment, although the lowest eGFR strata of MRAs and triple therapy contained the null in the 95% confidence interval. Results were similar for patients with HFmrEF (online supplementary *Figure* *S4B*).

### Re‐initiation of treatments

Among patients who stopped treatment, those with lower kidney function were less likely to re‐initiate them. In HFrEF, for ACEi/ARB/ARNi 82%, 78%, 71%, and 58% of patients restarted after discontinuation for eGFR ≥60, 45–59, 30–44, and <30 ml/min/1.73 m^2^, respectively (online supplementary *Table* *S4*). For beta‐blockers, this was 73%, 72%, 72%, and 65%; for MRAs 49%, 43%, 36%, and 27%; and for triple therapy 60%, 53%, 42%, and 28%. After multivariable analysis, patients with CKD still had lower odds of restarting treatment, except for beta‐blockers. Similar patterns were observed in patients with HFmrEF, in addition to lower odds for restarting beta‐blockers (online supplementary *Table* *S5*).

### Patterns of use of angiotensin receptor–neprilysin inhibitors in heart failure with reduced ejection fraction

Between January 2016 and December 2018, 13%, 13%, 10%, and 6% of HFrEF patients were prescribed ARNi for eGFR ≥60, 45–59, 30–44, and <30 ml/min/1.73 m^2^, respectively (online supplementary *Table* *S6*). Prescriptions were filled by 14%, 14%, 11%, and 7% of patients. The odds of being prescribed ARNi and the odds of filling a prescription for ARNi remained lower for patients with CKD after multivariable adjustment. As much as 84%, 80%, 63%, and 55% had low adherence and 63%, 59%, 48%, and 43% had low persistence for eGFR ≥60, 45–59, 30–44, and <30 ml/min/1.73 m^2^, respectively (online supplementary *Tables* *S7* and *S8*). After discontinuation, 82%, 84%, 79%, and 86% of patients restarted (online supplementary *Table* *S9*). The lower odds for patients with CKD of prescription and filling prescriptions persisted after multivariable adjustment, as did the lower odds of low adherence and low persistence.

When stratifying by the periods in which each ESC guideline was issued, we observed similar results to our main analyses, suggesting persistent suboptimal management of patients with CKD both in HFrEF (online supplementary *Figures* *S5–S7*) and in HFmrEF (online supplementary *Figures* *S8–S10*). In patients with HF and concomitant atrial fibrillation, the percentage of patients prescribed anticoagulants decreased with lower kidney function. For eGFR ≥60, 45–59, 30–44, and <30 ml/min/1.73 m^2^, the proportions were 79%, 79%, 75%, and 66%, respectively for HFrEF; and 79%, 78%, 77%, and 66%, respectively for HFmrEF (online supplementary *Table* *S1*), suggesting that undermanagement expands to other comorbidities of HF.

## Discussion

About half of patients with HF have concurrent CKD,^1^, ^2^ and this study evaluated in‐depth aspects of their pharmacological care. Patients with CKD were less likely to be prescribed and to fill prescriptions for RAASi/ARNi. In addition, they were less likely to adhere to all HFrEF guideline‐recommended medication and persist in RAASi/ARNi. Among patients who stopped medications, those with CKD were less likely to be restarted on them (*Graphical Abstract*). Collectively, this study provides evidence of suboptimal care in multiple dimensions of pharmacological treatment of this large segment of the HF population, which may in part explain their poor clinical outcomes.^3^, ^4^

In agreement with two US studies,^1^, ^18^ we report lower prescription rates among patients with CKD, expanding this finding to European settings with universal healthcare access. Although fear for adverse events (i.e. hyperkalaemia, worsening of kidney function)^5^ and contraindications may have impacted ACEi/ARB/ARNi or MRA prescriptions in advanced CKD (eGFR <30 ml/min/1.73 m^2^), we note that there are no clear contraindications for these medications in less severe CKD stages.^1^, ^6^, ^11^, ^12^, ^13^ However, the novelty of our analysis resides in the evaluation of the subsequent steps in the pharmacological management of these patients. Prescription of medications is important, but fruitless if the patient does not fill the prescription (i.e. initiate treatment), adhere to the treatment (i.e. dispense the drug according to the prescription), or persist on it (i.e. does not stop using these lifesaving medications).

We found that overall about 10% of patients fail to fill their prescriptions, and that patients with CKD are less likely to fill their prescription for all guideline‐recommended medical therapies except for beta‐blockers. Given that Sweden offers universal healthcare, and that the cost of medications is almost completely covered by the government, it is possible that compliance may be worse in countries with non‐subsidized health systems. We also found that patients with CKD were less likely to adhere to and persist in their treatments during the first year of therapy, particularly for RAASi/ARNi. Landmark trials^19^, ^20^, ^21^ showed these therapies to be effective also in patients with HF and CKD. However, a more common occurrence of hyperkalaemia events in patients with CKD^22^, ^23^ may cause patients to adhere less intensively to treatment or physicians to interrupt it. Routine‐care studies reveal that patients with CKD are indeed more likely to permanently interrupt their MRA treatment after hyperkalaemia,^22^, ^23^ despite potential benefits of continuing with these medications,^24^ as recommended by guidelines. Besides hyperkalaemia, worsening renal function is another important reason for stopping RAASi and MRA,^25^, ^26^, ^27^ although worsening renal function events may be defined in broad ways and may not necessarily lead to worse clinical outcomes.^25^, ^28^, ^29^, ^30^ Higher LVEF and worse NYHA class have been described as additional factors associated with stopping MRAs.^26^ Reasons for this potentially suboptimal care warrant investigation. In a Swiss study of patients with acute coronary syndrome, patients who discontinued medication tended to do so based on their physician's decision, while side effects, perceptions of the drug being unnecessary, and costs, were less likely to play a role in treatment interruptions.^31^

When evaluating patterns of use of the more recently available ARNi, we found overall a low implementation of use which might be at least partially explained by the recent introduction of this drug, but also by the required achievement of target dose of ACEi/ARB before switching to ARNi in the 2016 ESC guidelines on HF.^13^, ^32^ We also observed that patients with CKD were less likely to be prescribed and to fill prescriptions for them, which is consistent with the overall pattern of suboptimal treatment for these patients. However, we observed better adherence and persistence. Although a low sample size may provide unreliable estimates, a similar adherence/persistence (and even better in some strata) may be attributed to a lower occurrence of adverse events (i.e. rise in creatinine or hyperkalaemia) compared to traditional ACEi or MRAs.^33^ A US study found an overall acceptable adherence to ARNi in HF, although these patients were not all recently hospitalized and the authors did not have information on LVEF or kidney function.^34^

Over time, the use of guideline‐recommended therapies in HF has been increasing. A previous analysis in the SwedeHF Registry showed a gradual increase in the use of RAASi/ARNi, beta‐blockers, and MRAs in HFrEF^35^ and the 2021 annual SwedeHF Registry report also described an increased proportion of patients treated with triple therapy over the past few years.^36^ Nonetheless, underuse of MRA and limited implementation of ARNi remain a concern. The recent introduction of SGLT2i in the HFrEF guidelines, together with the positive results of SGLT2i trials in HFmrEF/HFpEF trials, highlights the need to developing implementation strategies to ensure the uptake of these recommendations. Implementing referral to HF specialist care has shown to positively affect prescription rates of guideline‐recommended medications.^37^ Educational opportunities for primary care physicians on clinical updates in HF and its management, and on how to face side effects and tolerance issues might also contribute to foster implementation of novel therapies and lower the risk of patients having life‐saving treatments interrupted. Use of potassium binders, as suggested by current guidelines,^6^ may help alleviate prescribers' fear of hyperkalaemia during treatment with MRA, ARNi and RAASi, which may be of particular concern among persons with compromised kidney function. Moreover, concomitant SGLT2i use in HFrEF, as recommended by guidelines,^6^ may also lower the risk of hyperkalaemia.^38^ Finally, implementing nationwide registries might allow screening strategies to identify undertreated patients and refer them to specialist care. Similar approaches might involve the use of electronic decision support systems providing protocols and checklists to remind clinicians of steps to follow during the patient's workup.

### Strengths and limitations

Our study has several strengths, including its large sample size, and accurate information on all aspects of medication prescription that allowed us to evaluate novel dimensions in treatment patterns with unprecedented granularity. We recognize that collecting dispensations at the pharmacy may not necessarily mean that the pills are being ingested. Further, it is not possible to dissect which actions depend on the physicians and which on the patients (i.e. we cannot ascertain whether the medication was not initiated because of patient behaviour/lack of compliance or because the prescriber changing their initial decision). Regardless, we believe these outcomes represent suboptimal treatment amenable to correction. Our assessment of kidney function is based on a single creatinine measurement at registration and may misclassify patients, but we argue this is the information available to physicians to prescribe and initiate treatments. We acknowledge the possibility that not all prescriptions might be registered in SwedeHF, as it depends on physicians reporting them, but believe this reporting bias to be likely small and proportional across eGFR strata. The information of pharmacy fills is however complete and collected by the government at a national level: prescriptions can only be dispensed at Swedish pharmacies through each citizen's personal identification number. Some covariates like body mass index or smoking had more than 10% of missingness and we were not able to control for them in our models. With the possibility of residual confounding in this and all observational analyses, causality cannot be inferred. Results represent Swedish clinical practice for 2009–2018, and extrapolation to other periods, countries or health systems needs to be done with caution. We were unable to evaluate use of SGLT2i, which was not approved in Sweden at the time of data collection. Not all patients with HF in Sweden are registered in SwedeHF, and we have previously shown that patients undertaking our registration protocols are more likely to receive guideline‐recommended therapies than those not registered.^39^ We thus speculate that the differences observed in our study may be larger at a national level.

## Conclusions

Patients with HF and CKD were not optimally treated with evidence‐based medical therapies, also at levels of eGFR where such therapies would not be contraindicated. The combined effects of even small deviations in each dimension of treatment (prescription, compliance, adherence, and persistence) may have important implications on outcomes. Because a lower provision of recommended therapies to persons with CKD has also been reported for patients with acute coronary syndrome^40^ or atrial fibrillation,^41^ this evidence collectively illustrates insecurity in how to manage this vulnerable group of patients.

### Funding

Research reported in this publication was supported by the Swedish Research Council (#2019–01059), the Swedish Heart and Lung Foundation and the Westman Foundation. R.J.J. is supported by grants from the Minerva Scholarship Fund and the Leiden University Fund (LUF) International Study Fund (LISF). E.L.F. acknowledges support by a Rubicon Grant of the Netherlands Organization for Scientific Research (NWO). The funders of this study had no role in the study design, data collection, analysis, interpretation, writing of the report, or the decision to submit the report for publication.

**Conflict of interest**: U.D. reports research grants from AstraZeneca, Vifor Pharma, Pfizer, Boehringer Ingelheim, Boston Scientific and Roche Diagnostics and consultancies/speaker honoraria from AstraZeneca, Pfizer and Amgen, all outside the submitted manuscript. J.J.C. reports consultation/speaker fees, advisory board membership or research funding from AstraZeneca, Viforpharma, Astellas, Bayer, Abbott, Baxter, Abbott, Fresenius Kabi and MSD, all outside the submitted work. G.S. reports grants and personal fees from Vifor, AstraZeneca, grants from Boston Scientific, Boehringer Ingelheim, Novartis, PHARMACOSMOS, Merck, Bayer, personal fees from Società Prodotti Antibiotici, Roche, Servier, GENESIS, Cytokinetics, Medtronic, outside the submitted work. All other authors have nothing to disclose.

## Supporting information


**Appendix S1**. Supplemental Information.
