# Supplementary material for: Use of guideline‐recommended medical therapy in patients with heart failure and chronic kidney disease: from physician's prescriptions to patient's dispensations, medication adherence and persistence
Source: Eur J Heart Fail. 2022 Aug 2;24(11):2185–95. doi: 10.1002/ejhf.2620 (PMC10087537; doi:10.1002/ejhf.2620)
Supplement: Supplementary file 1 — Appendix S1. Supplemental Information. [file EJHF-24-2185-s001.docx]

**Use of guideline-recommended medical therapy in patients with heart failure and chronic kidney disease: from physician’s prescriptions to patient’s dispensations, medication adherence and persistence**

*Supplementary materials*

Roemer J. Janse, Edouard L. Fu, Ulf Dahlström, Lina Benson, Bengt Lindholm, Merel van Diepen, Friedo W. Dekker, Lars H. Lund, Juan-Jesus Carrero, Gianluigi Savarese.

[Supplemental tables 3](#_Toc104897172)

[**Supplemental table 1.** Overview of treatment recommendations from the European Society of Cardiology guidelines for 2008, 2012, 2016, and 2021. 3](#_Toc104897173)

[**Supplemental table 2.** Definitions of covariates and outcomes. 4](#_Toc104897174)

[**Supplemental table 3.** Baseline characteristics for patients with heart failure with mildly reduced ejection fraction (HFmrEF), overall and by estimated glomerular filtration rate (eGFR, in ml/min/1.73 m2) categories. 5](#_Toc104897175)

[**Supplemental table 4.** Percentage of restarting within 3 months after discontinuation in the first 12 months after index among patients with heart failure with reduced ejection fraction (HFrEF). 7](#_Toc104897176)

[**Supplemental table 5.** Percentage of restarting within 3 months after discontinuation in the first 12 months after index among patients with heart failure with mildly reduced ejection fraction (HFmrEF). 8](#_Toc104897177)

[**Supplemental table 6.** Percentages of prescriptions and filled prescriptions for angiotensin receptor-neprilysin inhibitors among patients with heart failure with reduced ejection fraction (HFrEF). 9](#_Toc104897178)

[**Supplemental table 7.** Percentage of patients with low adherence in the first 12 months after index among patients using angiotensin receptor-neprilysin inhibitor for heart failure with reduced ejection fraction (HFrEF). 10](#_Toc104897179)

[**Supplemental table 8.** Percentage of patients with low persistence (i.e., discontinuation) in the first 12 months after index for angiotensin receptor-neprilysin inhibitors among patients with heart failure with reduced ejection fraction (HFrEF). 11](#_Toc104897180)

[**Supplemental table 9.** Percentage of restarting within 3 months after discontinuation in the first 12 months after index for angiotensin receptor-neprilysin inhibitors among patients with heart failure with reduced ejection fraction (HFrEF). 12](#_Toc104897181)

[**Supplemental table 10.** Percentages of prescriptions anticoagulants among atrial fibrillation patients with heart failure with reduced ejection fraction (HFrEF) or heart failure with mildly reduced ejection fraction (HFmrEF). 13](#_Toc104897182)

[Supplemental figures 14](#_Toc104897183)

[**Supplemental figure 1.** Flowchart of patient inclusion. 14](#_Toc104897184)

[**Supplemental figure 2.** Percentages of issued prescriptions at register entry (light bars) and filled prescriptions within 90 days from index date (dotted dark bars) by eGFR strata among patients with heart failure with mildly reduced ejection fraction (HFmrEF) in the Swedish Heart Failure register. 15](#_Toc104897185)

[**Supplemental figure 3.** Multivariable adjusted odds ratios (and 95% confidence intervals) for being prescribed and filling the prescription of guideline-recommended therapies in patients with heart failure with mildly reduced ejection fraction (HFmrEF) and differing eGFR categories. 16](#_Toc104897186)

[**Supplemental figure 4A.** Multivariable adjusted odds ratios (and 95% confidence intervals) for low adherence (proportion of days covered <80%) to guideline-recommended therapies during the first year of therapy in patients with heart failure with mildly reduced ejection fraction (HFmrEF) and differing eGFR categories. 17](#_Toc104897188)

[**Supplemental figure 4B.** Absolute risks and multivariable adjusted odds ratios (with 95% confidence intervals) for non-persistence (i.e. treatment discontinuation) to guideline-recommended therapies during the first year of therapy in patients with heart failure with mildly reduced ejection fraction (HFmrEF) and differing eGFR categories. 18](#_Toc104897189)

[**Supplemental figure 5.** Multivariable adjusted odds ratios (and 95% confidence intervals) for being prescribed guideline-recommended therapies in patients with heart failure with reduced ejection fraction (HFrEF) and differing eGFR categories in the time periods 2009-2011, 2012-2015, and 2016-2018. 19](#_Toc104897190)

[**Supplemental figure 6.** Multivariable adjusted odds ratios (and 95% confidence intervals) for filling prescriptions of guideline-recommended therapies in patients with heart failure with reduced ejection fraction (HFrEF) and differing eGFR categories in the time periods 2009-2011, 2012-2015, and 2016-2018. 20](#_Toc104897192)

[**Supplemental figure 7A.** Multivariable adjusted odds ratios (and 95% confidence intervals) for low adherence (proportion of days covered <80%) to guideline-recommended therapies during the first year of therapy in patients with heart failure with reduced ejection fraction (HFrEF) and differing eGFR categories in the time periods 2009-2011, 2012-2015, and 2016-2018. 21](#_Toc104897193)

[**Supplemental figure 7B.** Multivariable adjusted odds ratios (and 95% confidence intervals) for low persistence (i.e. treatment discontinuation) to guideline-recommended therapies during the first year of therapy in patients with heart failure with reduced ejection fraction (HFrEF) and differing eGFR categories in the time periods 2009-2011, 2012-2015, and 2016-2018. 22](#_Toc104897195)

[**Supplemental figure 8.** Multivariable adjusted odds ratios (and 95% confidence intervals) for being prescribed guideline-recommended therapies in patients with heart failure with mildly reduced ejection fraction (HFmrEF) and differing eGFR categories in the time periods 2009-2011, 2012-2015, and 2016-2018. 23](#_Toc104897197)

[**Supplemental figure 9.** Multivariable adjusted odds ratios (and 95% confidence intervals) for filling prescriptions of guideline-recommended therapies in patients with heart failure with mildly reduced ejection fraction (HFmrEF) and differing eGFR categories in the time periods 2009-2011, 2012-2015, and 2016-2018. 24](#_Toc104897198)

[**Supplemental figure 10A.** Multivariable adjusted odds ratios (and 95% confidence intervals) for low adherence (proportion of days covered <80%) to guideline-recommended therapies during the first year of therapy in patients with heart failure with mildly reduced ejection fraction (HFmrEF) and differing eGFR categories in the time periods 2009-2011, 2012-2015, and 2016-2018. 25](#_Toc104897199)

[**Supplemental figure 10B.** Multivariable adjusted odds ratios (and 95% confidence intervals) for low persistence (i.e. treatment discontinuation) to guideline-recommended therapies during the first year of therapy in patients with heart failure with mildly reduced ejection fraction (HFmrEF) and differing eGFR categories in the time periods 2009-2011, 2012-2015, and 2016-2018.](#_Toc104897200)

Supplemental tables

Supplemental table 1. Overview of treatment recommendations from the European Society of Cardiology guidelines for 2008, 2012, 2016, and 2021.

|  | **Reduced ejection fraction** | **Mildly reduced ejection fraction** | **Considerations for kidney function** |
| --- | --- | --- | --- |
| **ESC guidelines 2008** | | |  |
| ACEi/ARB | Recommended (I-A/I-B) | *HFmrEF was not yet a separate diagnosis in the 2008 guidelines. Recommendations were given for HFpEF, for which no treatment had been shown to convincingly reduce mortality.* | Contraindication for serum creatinine >220 µmol/L |
| Beta-blockers | Recommended (I-A) |  |  |
| MRA | Recommended (I-B) if signs and symptoms persist |  | Contraindication for serum creatinine >220 µmol/L |
| ARNi | - |  |  |
| Triple therapy | Recommended |  |  |
| **ESC guidelines 2012** | | |  |
| ACEi/ARB | Recommended (I-A) | *HFmrEF was not yet a separate diagnosis in the 2012 guidelines. Recommendations were given for HFpEF, for which no treatment had been shown to convincingly reduce mortality.* | Contraindication for eGFR <30 mL/min/1.73 m^2^ |
| Beta-blockers | Recommended (I-A) |  |  |
| MRA | Recommended (I-A) if signs and symptoms persist |  | Should only be used in patients with adequate renal function |
| ARNi | - |  |  |
| Triple therapy | Recommended |  |  |
| **ESC guidelines 2016** | | |  |
| ACEi/ARB | Recommended (I-A) | *HFmrEF was a separate diagnosis in the 2016 guidelines, but no treatment had been shown to convincingly reduce mortality.* | Caution/seek specialist advice for eGFR <30 mL/min/1.73 m^2^ |
| Beta-blockers | Recommended (I-A) |  |  |
| MRA | Recommended (I-A) if signs and symptoms persist |  | Caution/seek specialist advice for eGFR <30 mL/min/1.73 m^2^ |
| ARNi | Recommended (I-B) |  |  |
| Triple therapy | Recommended |  |  |
| **ESC guidelines 2021** | | |  |
| ACEi/ARB | Recommended (I-A/I-B) | May be considered (IIb-C) | Caution/seek specialist advice for eGFR <30 mL/min/1.73 m^2^ |
| Beta-blockers | Recommended (I-A) | May be considered (IIb-C) |  |
| MRA | Recommended (I-A) | May be considered (IIb-C) | Caution/seek specialist advice for eGFR <30 mL/min/1.73 m^2^ |
| ARNi | Recommended (I-A) | May be considered (IIb-C) | Contraindication for eGFR <30 mL/min/1.73 m^2^ |
| Triple therapy | Recommended | May be considered |  |

Recommendation and evidence are portrayed as (Class of recommendation-Level of evidence). Class of recommendation: I, recommended/indicated; IIa, should be considered; IIb, may be considered; III, not recommended. Levels of evidence: A, multiple randomized clinical trials or meta-analyses; B, single randomized clinical trial or large non-randomized studies; C, expert opinion consensus or small studies/retrospective studies/registers.

ESC, European Society of Cardiology; ACEi, angiotensin-converting enzyme inhibitor; ARB, angiotensin receptor blocker; MRA, mineralocorticoid receptor antagonist; ARNi, angiotensin receptor-neprilysin inhibitor; HFmrEF, heart failure with mildly reduced ejection fraction; HFpEF, heart failure with preserved ejection fraction; eGFR, estimated glomerular filtration rate.

Supplemental table 2. Definitions of covariates and outcomes.

| **Variable** | **Definition** |
| --- | --- |
| **Covariates** | |
| Coronary revascularization | As reported in register |
| Devices (CRT, ICD, or pacemaker) | As reported in register |
| Digoxin | As reported in register |
| Diuretics | As reported in register |
| Statins | As reported in register |
| Anticoagulants | As reported in register |
| Antiplatelets | As reported in register |
| Nitrates | As reported in register |
| Income | Above or below median of the whole register |
| Obesity | Body mass index > 30 kg/m^2^ |
| Atrial fibrillation | As reported in register + [ICD-10] I48 in the last 5 years |
| Anemia | Hemoglobin < 130 g/L for men or < 120 g/L for women |
| Cerebrovascular disease | As reported in register + [ICD-9] 430-434, 438; [ICD-10] I60-I64, I69.0-I69.4 + G45 in the last 5 years |
| COPD | [ICD-10] J40-J44 in the last 5 years |
| Dilated cardiomyopathy | [ICD-10] I42.0 in the last 5 years |
| Diabetes mellitus | As reported in register + [ICD-10] E10-E14 in the last 5 years |
| Hypertension | As reported in register + [ICD-10] I10-I15 in the last 5 years |
| Ischemic heart disease | [ICD-9] 410-414; [ICD-10] I20-I25 |
| Liver disease | [ICD-10] B18, I85, I86.4, I98.2, K70, K70.0, K71.1, K71.3-K71.7, K72-K74, K76.0, K76.2-K76.9 in the last 5 years |
| Peripheral artery disease | [ICD-10] I70-I73 in the last 5 years |
| Valvular disease | As reported in register + [ICD-10] I05-08, I34-I39, Q22, Q23.0-Q23.3, Z95.2-Z95.4 in the last 5 years |
| Cancer | [ICD-10] C in the last 3 years |
| **Outcomes** | |
| Prescription | As reported in register |
| Filled prescription | Entry in national prescription register (i.e., dispensed medication) within 60 days after index date |
| Adherence | Proportion of days covered <80% within the first 12 months after index date, taking into account stockpiling |
| Persistence | 60 subsequent days without pill supply within the first 12 months after index date, taking into account stockpiling |

CRT, cardiac resynchronization therapy; ICD, implantable cardioverter defibrillator; COPD, chronic obstructive pulmonary disease; ICD-10, International Classification of Diseases 10; ICD-9, International Classification of Diseases 9.

Supplemental table 3. Baseline characteristics for patients with heart failure with mildly reduced ejection fraction (HFmrEF), overall and by estimated glomerular filtration rate (eGFR, in mL/min/1.73 m2) categories.

|  | **% of missing** | **Overall** | **eGFR ≥60** | **eGFR 45-59** | **eGFR 30-44** | **eGFR <30** |
| --- | --- | --- | --- | --- | --- | --- |
| **Number of patients, n (%)** |  | 15,114 (100%) | 8,159 (54%) | 3,294 (22%) | 2,550 (17%) | 1,111 (7%) |
| **Age, median (IQR), y** |  | 76 [67, 83] | 71 [62, 78] | 80 [73, 84] | 82 [76, 86] | 82 [75, 87] |
| **Age group, n (%)^*^** |  |  |  |  |  |  |
| <45 years |  | 350 (2) | 327 (4) | 11 (0) | 3 (0) | 9 (1) |
| 46-65 |  | 2,833 (19) | 2,383 (29) | 236 (7) | 130 (5) | 84 (8) |
| 66-75 |  | 4,221 (28) | 2,709 (33) | 830 (25) | 480 (19) | 202 (18) |
| >75 |  | 7,710 (51) | 2,740 (34) | 2,217 (67) | 1,937 (76) | 816 (73) |
| **Women, %** |  | 5,645 (37) | 2,596 (32) | 1,340 (41) | 1,184 (46) | 525 (47) |
| **eGFR, median (IQR), mL/min/1.73m2**^*^ |  | 63 [46, 81] | 79 [69, 89] | 53 [49, 56] | 39 [35, 42] | 24 [19, 27] |
| **Smoking, n (%)^*^** | 23.4% |  |  |  |  |  |
| Never |  | 5,287 (46) | 2,655 (42) | 1,227 (49) | 1,018 (53) | 387 (47) |
| Former |  | 5,094 (44) | 2,769 (44) | 1,121 (44) | 826 (43) | 378 (46) |
| Current |  | 1,195 (10) | 869 (14) | 176 (7) | 95 (5) | 55 (7) |
| **Index year 2014-2018, %** |  | 9,329 (62) | 5,148 (63) | 2,007 (61) | 1,549 (61) | 625 (56) |
| **Other heart failure characteristics** |  |  |  |  |  |  |
| NYHA class III/IV, n (%)^*^ | 26.1% | 3,360 (30) | 1,344 (22) | 831 (35) | 784 (44) | 401 (55) |
| HF duration > 6 months, n (%) | 3.0% | 8,817 (60) | 4,183 (53) | 2,077 (65) | 1,772 (71) | 785 (73) |
| NT-proBNP, median (IQR), pg/mL^*^ | 39.8% | 1,596 [637, 3,568] | 1,010 [365, 2,255] | 2,006 [995, 3,870] | 2,889 [1,456, 5,864] | 5,612 [2,620, 12,274] |
| Hospitalized at register entry, n (%) |  | 4,656 (31) | 1,973 (24) | 1,087 (33) | 1,023 (40) | 573 (52) |
| **Comorbidities, n (%)** |  |  |  |  |  |  |
| Obesity^*^ | 44.9% | 2,330 (28) | 1,261 (28) | 470 (27) | 397 (28) | 202 (30) |
| Atrial fibrillation |  | 8,915 (59) | 4,187 (51) | 2,217 (67) | 1,789 (70) | 722 (65) |
| Anemia | 6.5% | 4,865 (34) | 1,882 (25) | 1,146 (37) | 1,124 (47) | 713 (67) |
| Cerebrovascular disease |  | 2,521 (17) | 1,095 (13) | 640 (19) | 541 (21) | 245 (22) |
| COPD |  | 2,080 (14) | 996 (12) | 490 (15) | 423 (17) | 171 (15) |
| Dilated cardiomyopathy |  | 1,361 (9) | 955 (12) | 222 (7) | 128 (5) | 56 (5) |
| Diabetes mellitus |  | 4,020 (27) | 1,846 (23) | 891 (27) | 814 (32) | 469 (42) |
| Hypertension |  | 10,623 (70) | 5,045 (62) | 2,555 (78) | 2,071 (81) | 952 (86) |
| Ischemic heart disease |  | 8,454 (56) | 4,167 (51) | 1,964 (60) | 1,603 (63) | 720 (65) |
| Liver disease | 1.5% | 2,585 (17) | 1,296 (16) | 614 (19) | 485 (19) | 190 (17) |
| Peripheral artery disease |  | 1,432 (9) | 616 (8) | 323 (10) | 310 (12) | 183 (16) |
| Valvular disease |  | 4,219 (28) | 1,877 (23) | 1,082 (33) | 855 (34) | 405 (36) |
| Cancer |  | 2,006 (13) | 975 (12) | 484 (15) | 373 (15) | 174 (16) |
| **Procedures, n (%)** |  |  |  |  |  |  |
| Coronary revascularization |  | 5,285 (35) | 2,717 (33) | 1,189 (36) | 951 (37) | 428 (39) |
| Devices (CRT, ICD, or pacemaker) | 1.8% | 2,249 (15) | 1,007 (13) | 563 (17) | 479 (19) | 200 (18) |
| **Medication, n (%)** |  |  |  |  |  |  |
| Digoxin | 0.3% | 1,840 (12) | 1,008 (12) | 456 (14) | 301 (12) | 75 (7) |
| Diuretics | 0.3% | 10,572 (70) | 4,647 (57) | 2,633 (80) | 2,269 (89) | 1,023 (93) |
| Statins | 0.3% | 7,802 (52) | 4,233 (52) | 1,687 (51) | 1,321 (52) | 561 (51) |
| Anticoagulants | 0.3% | 7,439 (49) | 3,638 (45) | 1,848 (56) | 1,438 (57) | 515 (47) |
| Antiplatelets | 0.4% | 5,951 (40) | 3,312 (41) | 1,232 (38) | 933 (37) | 474 (43) |
| Nitrates | 0.4% | 1,900 (13) | 694 (9) | 469 (14) | 475 (19) | 262 (24) |
| **Clinical measures** |  |  |  |  |  |  |
| BMI, median (IQR), kg/m^2*^ | 44.9% | 27 [24, 31] | 27 [24, 31] | 27 [24, 30] | 27 [23, 30] | 27 [23, 32] |
| Systolic BP, median (IQR), mmHg | 1.8% | 130 [115, 140] | 130 [115, 140] | 129 [115, 140] | 126 [114, 140] | 130 [115, 145] |
| Diastolic BP, median (IQR), mmHg | 1.8% | 72 [65, 80] | 75 [68, 80] | 70 [65, 80] | 70 [60, 80] | 70 [60, 80] |
| Mean arterial pressure, mean (SD), mmHg^*^ | 1.8% | 92 (13) | 93 (13) | 91 (13) | 90 (13) | 91 (13) |
| Hemoglobin, mean (SD), g/L | 6.5% | 132 (17) | 136 (17) | 130 (17) | 126 (17) | 118 (16) |
| Potassium, mean (SD), mmol/L^*^ | 9.4% | 4 (0) | 4 (0) | 4 (0) | 4 (0) | 4 (1) |
| Heart rate, median (IQR), BPM | 3.0% | 70 [62, 80] | 70 [61, 80] | 70 [62, 80] | 71 [62, 80] | 72 [64, 80] |
| **Socioeconomic characteristics, n (%)** |  |  |  |  |  |  |
| Single civil status | 0.1% | 7,027 (47) | 3,508 (43) | 1,573 (48) | 1,348 (53) | 598 (54) |
| Income above median |  | 8,827 (58) | 5,223 (64) | 1,788 (54) | 1,282 (50) | 534 (48) |
| Highest achieved education | 1.8% |  |  |  |  |  |
| Compulsory school |  | 6,272 (42) | 2,947 (37) | 1,506 (47) | 1,266 (51) | 553 (51) |
| Secondary school |  | 5,939 (40) | 3,484 (43) | 1,211 (37) | 865 (35) | 379 (35) |
| University |  | 2,636 (18) | 1,603 (20) | 518 (16) | 366 (15) | 149 (14) |

Categorical variables are presented as percentage. Continuous variables are presented as mean (standard deviation) or median [interquartile range], depending on the distribution.

eGFR, estimated glomerular filtration rate; IQR, interquartile range; NYHA, New York Heart Association functional classification; NT-proBNP, N-terminal prohormone of brain natriuretic peptide; COPD, chronic obstructive pulmonary disease; CRT, cardiac resynchronization therapy; ICD, implantable cardioverter defibrillator; BMI, body mass index; BP, blood pressure; SD, standard deviation; BPM, beats per minute.

^*^ Not adjusted for in analyses.

Supplemental table 4. Percentage of restarting within 3 months after discontinuation in the first 12 months after index among patients with heart failure with reduced ejection fraction (HFrEF).

| **eGFR strata** | **Number of patients** | **Restart** | **%** | **Univariable OR**  **(95% CI)** | **Multivariable OR^a^ (95% CI)** |
| --- | --- | --- | --- | --- | --- |
| **ACEi/ARB/ARNi** | | | | | |
| eGFR ≥60 mL/min/1.73m^2^ | 7,609 | 6,230 | 82 | 1 (Reference) | 1 (Reference) |
| eGFR 45-59 mL/min/1.73m^2^ | 3,039 | 2,358 | 78 | 0.77 (0.69-0.85) | 0.86 (0.77-0.97) |
| eGFR 30-44 mL/min/1.73m^2^ | 2,242 | 1,600 | 71 | 0.74 (0.70-0.78) | 0.84 (0.78-0.89) |
| eGFR <30 mL/min/1.73m^2^ | 822 | 476 | 58 | 0.67 (0.64-0.71) | 0.76 (0.71-0.80) |
| **Beta-blocker** | | | | | |
| eGFR ≥60 mL/min/1.73m^2^ | 2,890 | 2,109 | 73 | 1 (Reference) | 1 (Reference) |
| eGFR 45-59 mL/min/1.73m^2^ | 1,160 | 838 | 72 | 0.96 (0.83-1.12) | 0.98 (0.83-1.16) |
| eGFR 30-44 mL/min/1.73m^2^ | 872 | 626 | 72 | 0.97 (0.89-1.06) | 0.98 (0.89-1.08) |
| eGFR <30 mL/min/1.73m^2^ | 351 | 228 | 65 | 0.88 (0.82-0.95) | 0.93 (0.85-1.02) |
| **MRA** | | | | | |
| eGFR ≥60 mL/min/1.73m^2^ | 2,269 | 1,122 | 49 | 1 (Reference) | 1 (Reference) |
| eGFR 45-59 mL/min/1.73m^2^ | 952 | 412 | 43 | 0.78 (0.67-0.91) | 0.79 (0.67-0.94) |
| eGFR 30-44 mL/min/1.73m^2^ | 718 | 262 | 36 | 0.77 (0.70-0.84) | 0.81 (0.73-0.89) |
| eGFR <30 mL/min/1.73m^2^ | 169 | 45 | 27 | 0.72 (0.64-0.81) | 0.76 (0.67-0.87) |
| **Triple therapy** | | | | | |
| eGFR ≥60 mL/min/1.73m^2^ | 3,293 | 1,965 | 60 | 1 (Reference) | 1 (Reference) |
| eGFR 45-59 mL/min/1.73m^2^ | 1,203 | 639 | 53 | 0.77 (0.67-0.87) | 0.84 (0.73-0.97) |
| eGFR 30-44 mL/min/1.73m^2^ | 710 | 297 | 42 | 0.70 (0.64-0.76) | 0.77 (0.70-0.84) |
| eGFR <30 mL/min/1.73m^2^ | 161 | 45 | 28 | 0.64 (0.57-0.72) | 0.69 (0.61-0.78) |

eGFR, estimated glomerular filtration rate; OR, odds ratio; CI, confidence interval; ACEi, angiotensin-converting enzyme inhibitor; ARB, angiotensin receptor blocker; ARNi, angiotensin receptor-neprilysin inhibitor; MRA, mineralocorticoid receptor antagonist.

^a^ Adjusted for age, sex, hospitalization at index, heart failure duration, anemia, atrial fibrillation, cerebrovascular disease, chronic obstructive pulmonary disease, diabetes mellitus, dilated cardiomyopathy, hypertension, ischemic heart disease, liver disease, peripheral artery disease, valvular disease, cancer, coronary revascularization, devices (CRT, ICD, or pacemaker), prescription for digoxin, diuretics, statins, anticoagulants, antiplatelets, or nitrates at index, heart rate, systolic and diastolic blood pressure, hemoglobin, highest achieved education, civil status, income, and year of index category.

Supplemental table 5. Percentage of restarting within 3 months after discontinuation in the first 12 months after index among patients with heart failure with mildly reduced ejection fraction (HFmrEF).

| **eGFR strata** | **Number of patients** | **Restart** | **%** | **Univariable OR**  **(95% CI)** | **Multivariable OR^a^ (95% CI)** |
| --- | --- | --- | --- | --- | --- |
| **ACEi/ARB/ARNi** | | | | | |
| eGFR ≥60 mL/min/1.73m^2^ | 3,854 | 3,108 | 81 | 1 (Reference) | 1 (Reference) |
| eGFR 45-59 mL/min/1.73m^2^ | 1,464 | 1,118 | 76 | 0.78 (0.67-0.90) | 0.87 (0.74-1.02) |
| eGFR 30-44 mL/min/1.73m^2^ | 1,071 | 753 | 70 | 0.75 (0.70-0.81) | 0.82 (0.75-0.90) |
| eGFR <30 mL/min/1.73m^2^ | 394 | 237 | 60 | 0.71 (0.66-0.77) | 0.80 (0.74-0.87) |
| **Beta-blocker** | | | | | |
| eGFR ≥60 mL/min/1.73m^2^ | 1,630 | 1,180 | 72 | 1 (Reference) | 1 (Reference) |
| eGFR 45-59 mL/min/1.73m^2^ | 598 | 401 | 67 | 0.78 (0.63-0.95) | 0.75 (0.60-0.94) |
| eGFR 30-44 mL/min/1.73m^2^ | 451 | 295 | 65 | 0.85 (0.76-0.95) | 0.85 (0.75-0.97) |
| eGFR <30 mL/min/1.73m^2^ | 208 | 119 | 57 | 0.80 (0.72-0.88) | 0.81 (0.73-0.91) |
| **MRA** | | | | | |
| eGFR ≥60 mL/min/1.73m^2^ | 706 | 345 | 49 | 1 (Reference) | 1 (Reference) |
| eGFR 45-59 mL/min/1.73m^2^ | 368 | 156 | 42 | 0.77 (0.60-0.99) | 0.90 (0.66-1.22) |
| eGFR 30-44 mL/min/1.73m^2^ | 282 | 106 | 38 | 0.79 (0.69-0.91) | 0.87 (0.73-1.05) |
| eGFR <30 mL/min/1.73m^2^ | 85 | 19 | 22 | 0.67 (0.56-0.80) | 0.76 (0.62-0.93) |
| **Triple therapy** | | | | | |
| eGFR ≥60 mL/min/1.73m^2^ | 912 | 554 | 61 | 1 (Reference) | 1 (Reference) |
| eGFR 45-59 mL/min/1.73m^2^ | 421 | 225 | 53 | 0.74 (0.59-0.94) | 0.92 (0.70-1.21) |
| eGFR 30-44 mL/min/1.73m^2^ | 261 | 103 | 39 | 0.65 (0.56-0.75) | 0.76 (0.64-0.89) |
| eGFR <30 mL/min/1.73m^2^ | 57 | 13 | 23 | 0.58 (0.47-0.71) | 0.64 (0.51-0.81) |

eGFR, estimated glomerular filtration rate; OR, odds ratio; CI, confidence interval; ACEi, angiotensin-converting enzyme inhibitor; ARB, angiotensin receptor blocker; ARNi, angiotensin receptor-neprilysin inhibitor; MRA, mineralocorticoid receptor antagonist.

^a^ Adjusted for age, sex, hospitalization at index, heart failure duration, anemia, atrial fibrillation, cerebrovascular disease, chronic obstructive pulmonary disease, diabetes mellitus, dilated cardiomyopathy, hypertension, ischemic heart disease, liver disease, peripheral artery disease, valvular disease, cancer, coronary revascularization, devices (CRT, ICD, or pacemaker), prescription for digoxin, diuretics, statins, anticoagulants, antiplatelets, or nitrates at index, heart rate, systolic and diastolic blood pressure, hemoglobin, highest achieved education, civil status, income, and year of index category.

Supplemental table 6. Percentages of prescriptions and filled prescriptions for angiotensin receptor-neprilysin inhibitors among patients with heart failure with reduced ejection fraction (HFrEF).

| **eGFR strata^a^** | **Number of patients** | **Prescrip-tions** | **%** | **Univariable OR (95% CI)** | **Multivariable OR^b^ (95% CI)** | **Number of patients** | **Filled prescript-tions** | **%** | **Univariable OR (95% CI)** | **Multivariable OR^b^ (95% CI)** |
| --- | --- | --- | --- | --- | --- | --- | --- | --- | --- | --- |
| **HFrEF** | | | | | | | | | | |
| eGFR ≥60 | 6,795 | 863 | 13 | 1 (Reference) | 1 (Reference) | 6,795 | 943 | 14 | 1 (Reference) | 1 (Reference) |
| eGFR 45-59 | 2,822 | 380 | 13 | 1.07 (0.94-1.22) | 1.09 (0.93-1.27) | 2,822 | 391 | 14 | 1.00 (0.88-1.13) | 1.03 (0.89-1.20) |
| eGFR 30-44 | 2,023 | 204 | 10 | 0.88 (0.81-0.95) | 0.86 (0.78-0.94) | 2,023 | 217 | 11 | 0.86 (0.80-0.93) | 0.85 (0.78-0.94) |
| eGFR <30 | 754 | 45 | 6 | 0.76 (0.68-0.84) | 0.76 (0.68-0.86) | 754 | 51 | 7 | 0.77 (0.70-0.84) | 0.77 (0.69-0.86) |

eGFR, estimated glomerular filtration rate; OR, odds ratio; CI, confidence interval; HFrEF, heart failure with reduced ejection fraction.

^a^ in mL/min/1.73m^2^.

^b^ Adjusted for age, sex, hospitalization at index, heart failure duration, anemia, atrial fibrillation, cerebrovascular disease, chronic obstructive pulmonary disease, diabetes mellitus, dilated cardiomyopathy, hypertension, ischemic heart disease, liver disease, peripheral artery disease, valvular disease, cancer, coronary revascularization, devices (CRT, ICD, or pacemaker), prescription for digoxin, diuretics, statins, anticoagulants, antiplatelets, or nitrates at index, heart rate, systolic and diastolic blood pressure, hemoglobin, highest achieved education, civil status, and income.

Supplemental table 7. Percentage of patients with low adherence in the first 12 months after index among patients using angiotensin receptor-neprilysin inhibitor for heart failure with reduced ejection fraction (HFrEF).

| **eGFR strata** | **Number of patients** | **Low PDC** | **%** | **Univariable OR**  **(95% CI)** | **Multivariable OR^a^ (95% CI)** |
| --- | --- | --- | --- | --- | --- |
| **HFrEF** | | | | | |
| eGFR ≥60 mL/min/1.73m^2^ | 943 | 793 | 84 | 1 (Reference) | 1 (Reference) |
| eGFR 45-59 mL/min/1.73m^2^ | 391 | 311 | 80 | 0.74 (0.54-0.99) | 0.98 (0.70-1.39) |
| eGFR 30-44 mL/min/1.73m^2^ | 217 | 137 | 63 | 0.57 (0.48-0.67) | 0.68 (0.56-0.83) |
| eGFR <30 mL/min/1.73m^2^ | 51 | 28 | 55 | 0.61 (0.51-0.74) | 0.64 (0.50-0.81) |

eGFR, estimated glomerular filtration rate; PDC, proportion of days covered; OR, odds ratio; CI, confidence interval; HFrEF, heart failure with reduced ejection fraction.

^a^ Adjusted for age, sex, hospitalization at index, heart failure duration, anemia, atrial fibrillation, cerebrovascular disease, chronic obstructive pulmonary disease, diabetes mellitus, dilated cardiomyopathy, hypertension, ischemic heart disease, liver disease, peripheral artery disease, valvular disease, cancer, coronary revascularization, devices (CRT, ICD, or pacemaker), prescription for digoxin, diuretics, statins, anticoagulants, antiplatelets, or nitrates at index, heart rate, systolic and diastolic blood pressure, hemoglobin, highest achieved education, civil status, and income.

Supplemental table 8. Percentage of patients with low persistence (i.e., discontinuation) in the first 12 months after index for angiotensin receptor-neprilysin inhibitors among patients with heart failure with reduced ejection fraction (HFrEF).

| **eGFR strata** | **Number of patients** | **Discon-tinued** | **%** | **Univariable OR**  **(95% CI)** | **Multivariable OR^a^ (95% CI)** | **Absolute risk (95% CI)^b^** |
| --- | --- | --- | --- | --- | --- | --- |
| **HFrEF** | | | |  | | |
| eGFR ≥60 mL/min/1.73m^2^ | 943 | 598 | 63 | 1 (Reference) | 1 (Reference) | 64.8% (61.8-68.0) |
| eGFR 45-59 mL/min/1.73m^2^ | 391 | 232 | 59 | 0.84 (0.66-1.07) | 0.98 (0.75-1.29) | 61.9% (57.2-67.1) |
| eGFR 30-44 mL/min/1.73m^2^ | 217 | 104 | 48 | 0.73 (0.63-0.85) | 0.78 (0.66-0.93) | 52.3% (45.7-59.9) |
| eGFR <30 mL/min/1.73m^2^ | 51 | 22 | 43 | 0.76 (0.63-0.92) | 0.79 (0.63-0.98) | 50.5% (37.3-68.4) |

eGFR, estimated glomerular filtration rate; OR, odds ratio; CI, confidence interval; HFrEF, heart failure with reduced ejection fraction.

^a^ Adjusted for age, sex, hospitalization at index, heart failure duration, anemia, atrial fibrillation, cerebrovascular disease, chronic obstructive pulmonary disease, diabetes mellitus, dilated cardiomyopathy, hypertension, ischemic heart disease, liver disease, peripheral artery disease, valvular disease, cancer, coronary revascularization, devices (CRT, ICD, or pacemaker), prescription for digoxin, diuretics, statins, anticoagulants, antiplatelets, or nitrates at index, heart rate, systolic and diastolic blood pressure, hemoglobin, highest achieved education, civil status, and income.

^b^ The absolute risks takes into account the competing risk of death.

Supplemental table 9. Percentage of restarting within 3 months after discontinuation in the first 12 months after index for angiotensin receptor-neprilysin inhibitors among patients with heart failure with reduced ejection fraction (HFrEF).

| **eGFR strata** | **Number of patients** | **Restart** | **%** | **Univariable OR**  **(95% CI)** | **Multivariable OR^a^ (95% CI)** |
| --- | --- | --- | --- | --- | --- |
| **HFrEF** | | | | | |
| eGFR ≥60 mL/min/1.73m^2^ | 598 | 492 | 82 | 1 (Reference) | 1 (Reference) |
| eGFR 45-59 mL/min/1.73m^2^ | 232 | 195 | 84 | 1.14 (0.75-1.71) | 1.20 (0.75-1.90) |
| eGFR 30-44 mL/min/1.73m^2^ | 104 | 82 | 79 | 0.90 (0.69-1.16) | 1.08 (0.80-1.47) |
| eGFR <30 mL/min/1.73m^2^ | 22 | 19 | 86 | 1.11 (0.73-1.67) | 1.24 (0.79-1.94) |

eGFR, estimated glomerular filtration rate; OR, odds ratio; CI, confidence interval; HFrEF, heart failure with reduced ejection fraction.

^a^ Adjusted for age, sex, hospitalization at index, heart failure duration, anemia, atrial fibrillation, cerebrovascular disease, chronic obstructive pulmonary disease, diabetes mellitus, dilated cardiomyopathy, hypertension, ischemic heart disease, liver disease, peripheral artery disease, valvular disease, cancer, coronary revascularization, devices (CRT, ICD, or pacemaker), prescription for digoxin, diuretics, statins, anticoagulants, antiplatelets, or nitrates at index, heart rate, systolic and diastolic blood pressure, hemoglobin, highest achieved education, civil status, and income.

Supplemental table 10. Percentages of prescriptions and multivariable adjusted odds ratios for anticoagulants among atrial fibrillation patients with heart failure with reduced ejection fraction (HFrEF) or heart failure with mildly reduced ejection fraction (HFmrEF).

| **eGFR strata** | **Number of patients** | **Prescriptions** | **%** | **Univariable OR**  **(95% CI)** | **Multivariable OR^a^ (95% CI)** |
| --- | --- | --- | --- | --- | --- |
| **HFrEF** | | | | | |
| eGFR ≥60 mL/min/1.73m^2^ | 8,256 | 6,552 | 79 | 1 (Reference) | 1 (Reference) |
| eGFR 45-59 mL/min/1.73m^2^ | 4,160 | 3,272 | 79 | 0.96 (0.87-1.05) | 1.11 (0.97-1.26) |
| eGFR 30-44 mL/min/1.73m^2^ | 3,434 | 2,583 | 75 | 0.89 (0.85-0.93) | 1.03 (0.96-1.10) |
| eGFR <30 mL/min/1.73m^2^ | 1,401 | 929 | 66 | 0.80 (0.77-0.83) | 0.95 (0.90-1.02) |
| **HFmrEF** |  |  |  |  |  |
| eGFR ≥60 mL/min/1.73m^2^ | 4,175 | 3,299 | 79 | 1 (Reference) | 1 (Reference) |
| eGFR 45-59 mL/min/1.73m^2^ | 2,210 | 1,731 | 78 | 0.96 (0.85-1.09) | 1.03 (0.87-1.24) |
| eGFR 30-44 mL/min/1.73m^2^ | 1,780 | 1,379 | 77 | 0.96 (0.89-1.02) | 1.00 (0.91-1.11) |
| eGFR <30 mL/min/1.73m^2^ | 718 | 476 | 66 | 0.81 (0.76-0.85) | 0.85 (0.77-0.92) |

eGFR, estimated glomerular filtration rate; OR, odds ratio; CI, confidence interval; HFrEF, heart failure with reduced ejection fraction; HFmrEF, heart failure with mildly reduced ejection fraction.

^a^ Adjusted for age, sex, hospitalization at index, heart failure duration, anemia, atrial fibrillation, cerebrovascular disease, chronic obstructive pulmonary disease, diabetes mellitus, dilated cardiomyopathy, hypertension, ischemic heart disease, liver disease, peripheral artery disease, valvular disease, cancer, coronary revascularization, devices (CRT, ICD, or pacemaker), prescription for digoxin, diuretics, statins, anticoagulants, antiplatelets, or nitrates at index, heart rate, systolic and diastolic blood pressure, hemoglobin, highest achieved education, civil status, and income.

Supplemental figures

Supplemental figure 1. Flowchart of patient inclusion.


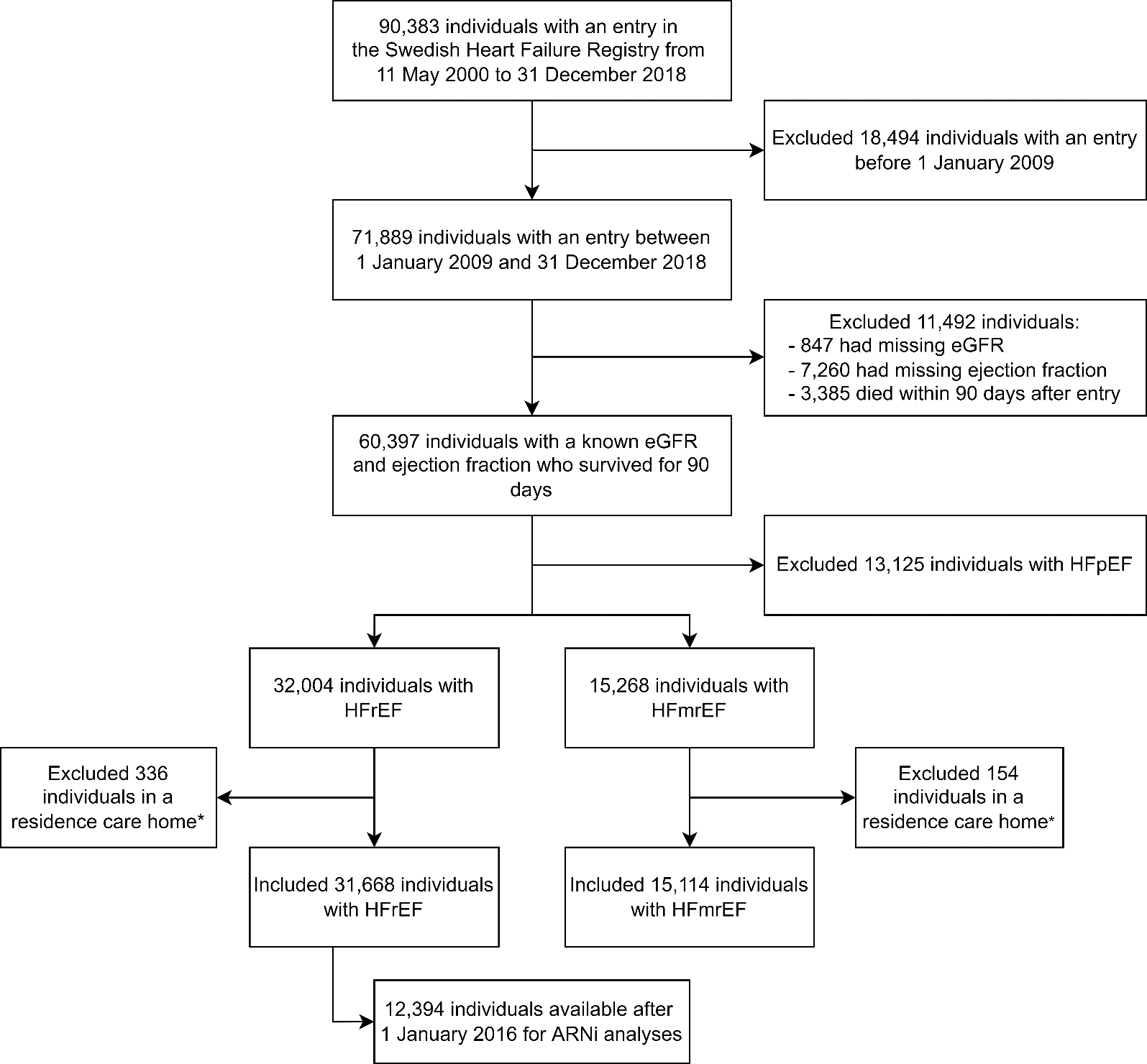


eGFR, estimated glomerular filtration rate; HFpEF, heart failure with preserved ejection fraction; HFrEF, heart failure with reduced ejection fraction; HFmrEF, heart failure with mildly reduced ejection fraction; ARNi, angiotensin receptor-neprilysin inhibitor.

* Information not available between 2010-04-09 and 2017-04-26.

Supplemental figure 2. Percentages of issued prescriptions at register entry (light bars) and filled prescriptions within 90 days from index date (dotted dark bars) by eGFR strata among patients with heart failure with mildly reduced ejection fraction (HFmrEF) in the Swedish Heart Failure register.


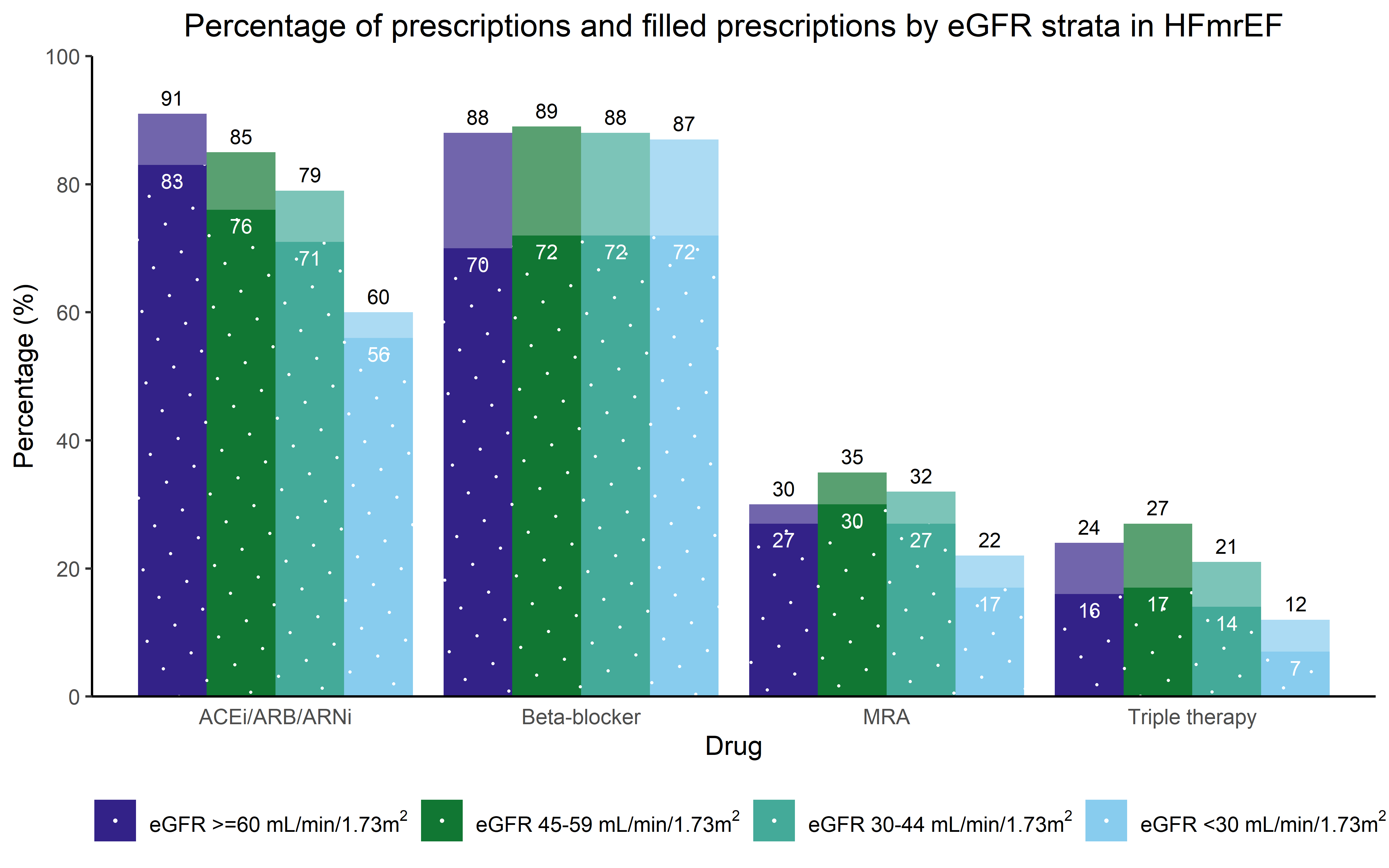


eGFR, estimated glomerular filtration rate; HFrEF, heart failure with reduced ejection fraction; ACEi, angiotensin-converting enzyme inhibitor; ARB, angiotensin receptor blocker; ARNi, angiotensin receptor-neprilysin inhibitor; MRA, mineralocorticoid receptor antagonist.

Supplemental figure 3. Multivariable adjusted odds ratios (and 95% confidence intervals) for being prescribed and filling the prescription of guideline-recommended therapies in patients with heart failure with mildly reduced ejection fraction (HFmrEF) and differing eGFR categories.


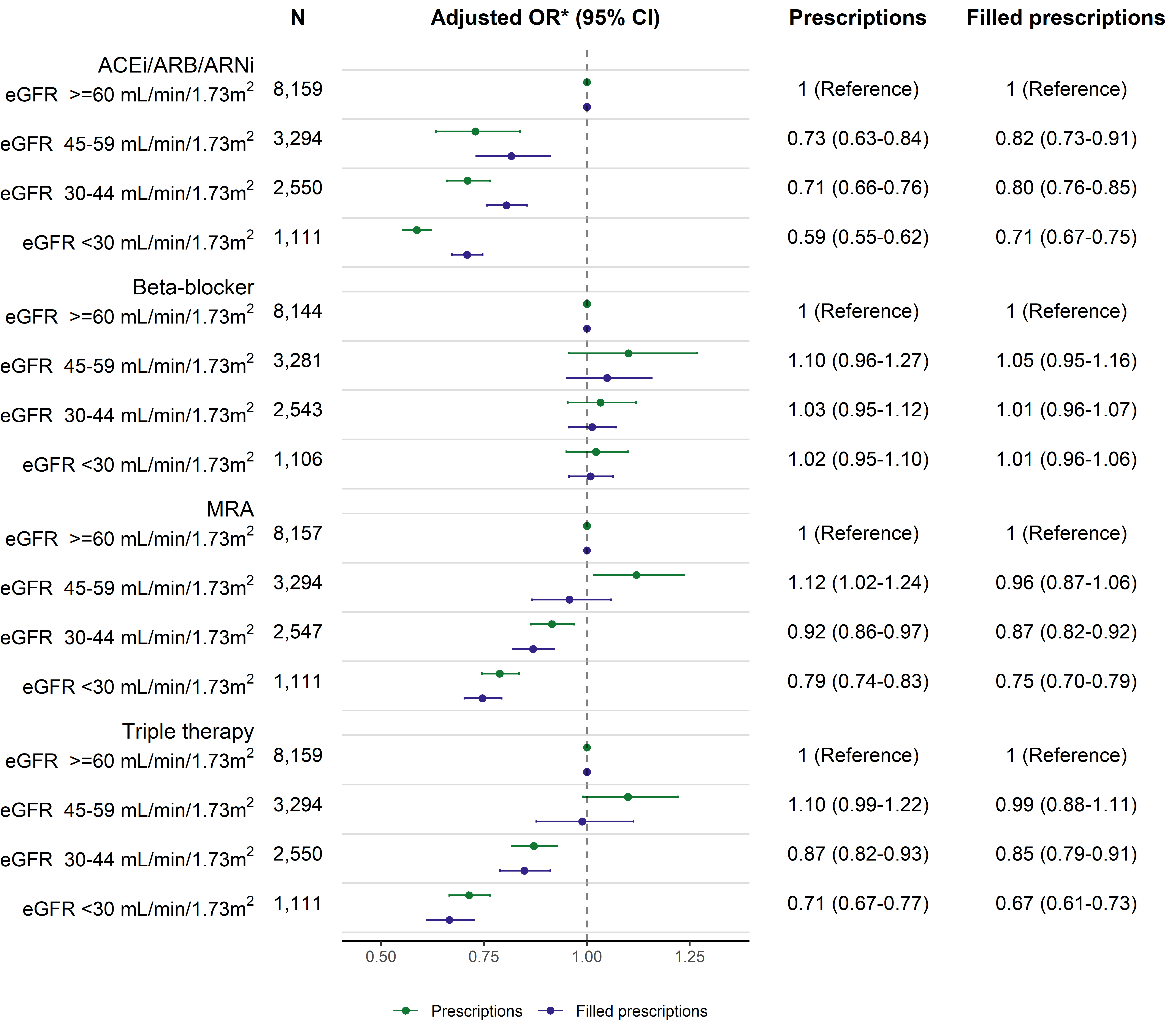


OR, odds ratio; CI, confidence interval; ACEi, angiotensin-converting enzyme inhibitor; ARB, angiotensin receptor blocker; ARNi, angiotensin receptor-neprilysin inhibitor; eGFR, estimated glomerular filtration rate, MRA, mineralocorticoid receptor antagonist.

^*^ Adjusted for age, sex, hospitalization at index, heart failure duration, anemia, atrial fibrillation, cerebrovascular disease, chronic obstructive pulmonary disease, diabetes mellitus, dilated cardiomyopathy, hypertension, ischemic heart disease, liver disease, peripheral artery disease, valvular disease, cancer, coronary revascularization, devices (CRT, ICD, or pacemaker), prescription for digoxin, diuretics, statins, anticoagulants, antiplatelets, or nitrates at index, heart rate, systolic and diastolic blood pressure, hemoglobin, highest achieved education, civil status, income, and year of index category.

Supplemental figure 4A. Multivariable adjusted odds ratios (and 95% confidence intervals) for low adherence (proportion of days covered <80%) to guideline-recommended therapies during the first year of therapy in patients with heart failure with mildly reduced ejection fraction (HFmrEF) and differing eGFR categories.


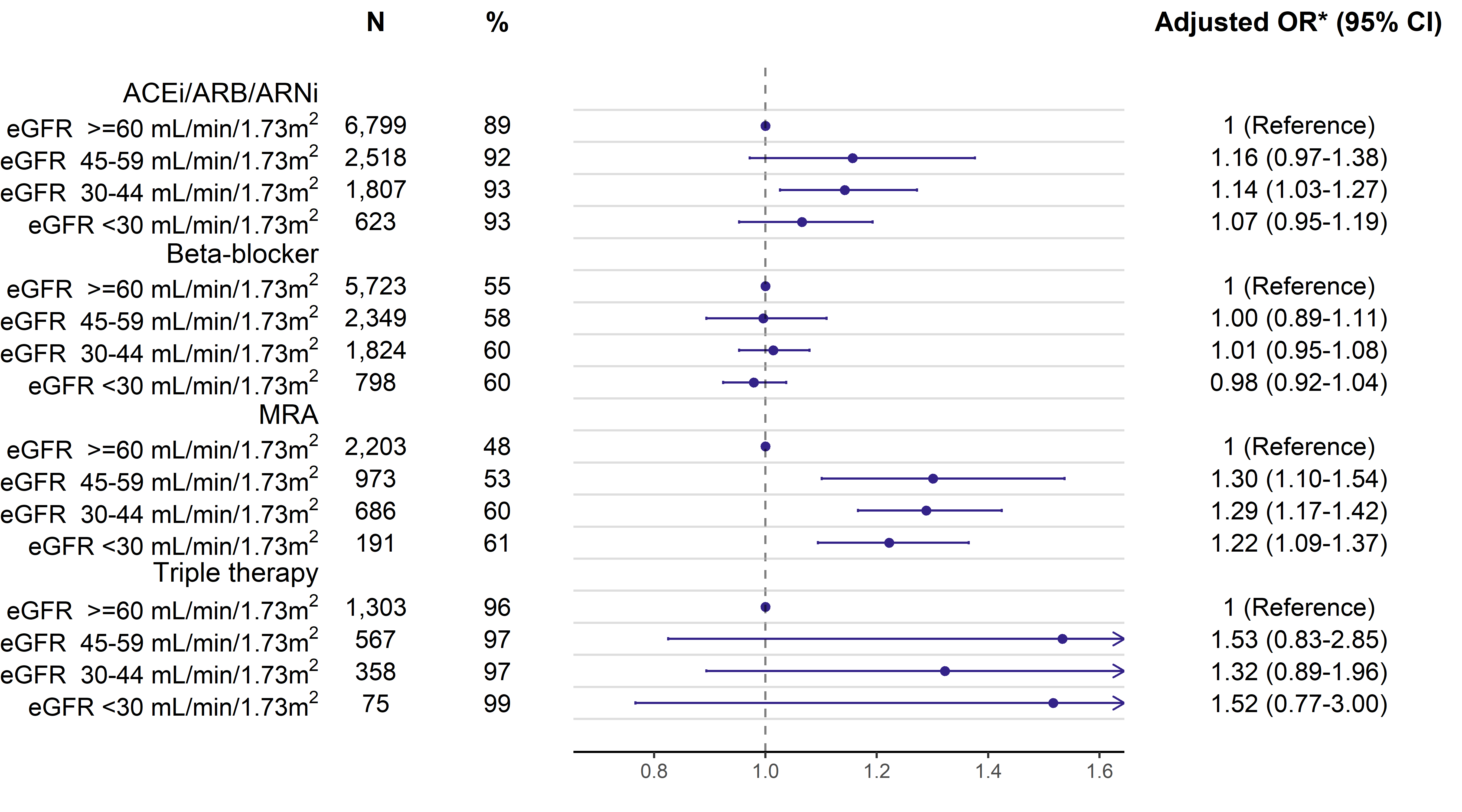


OR, odds ratio; CI, confidence interval; ACEi, angiotensin-converting enzyme inhibitor; ARB, angiotensin receptor blocker; ARNi, angiotensin receptor-neprilysin inhibitor; eGFR, estimated glomerular filtration rate, MRA, mineralocorticoid receptor antagonist.

^*^ Adjusted for age, sex, hospitalization at index, heart failure duration, anemia, atrial fibrillation, cerebrovascular disease, chronic obstructive pulmonary disease, diabetes mellitus, dilated cardiomyopathy, hypertension, ischemic heart disease, liver disease, peripheral artery disease, valvular disease, cancer, coronary revascularization, devices (CRT, ICD, or pacemaker), prescription for digoxin, diuretics, statins, anticoagulants, antiplatelets, or nitrates at index, heart rate, systolic and diastolic blood pressure, hemoglobin, highest achieved education, civil status, income, and year of index category.

Supplemental figure 4B. Absolute risks and multivariable adjusted odds ratios (with 95% confidence intervals) for non-persistence (i.e. treatment discontinuation) to guideline-recommended therapies during the first year of therapy in patients with heart failure with mildly reduced ejection fraction (HFmrEF) and differing eGFR categories.


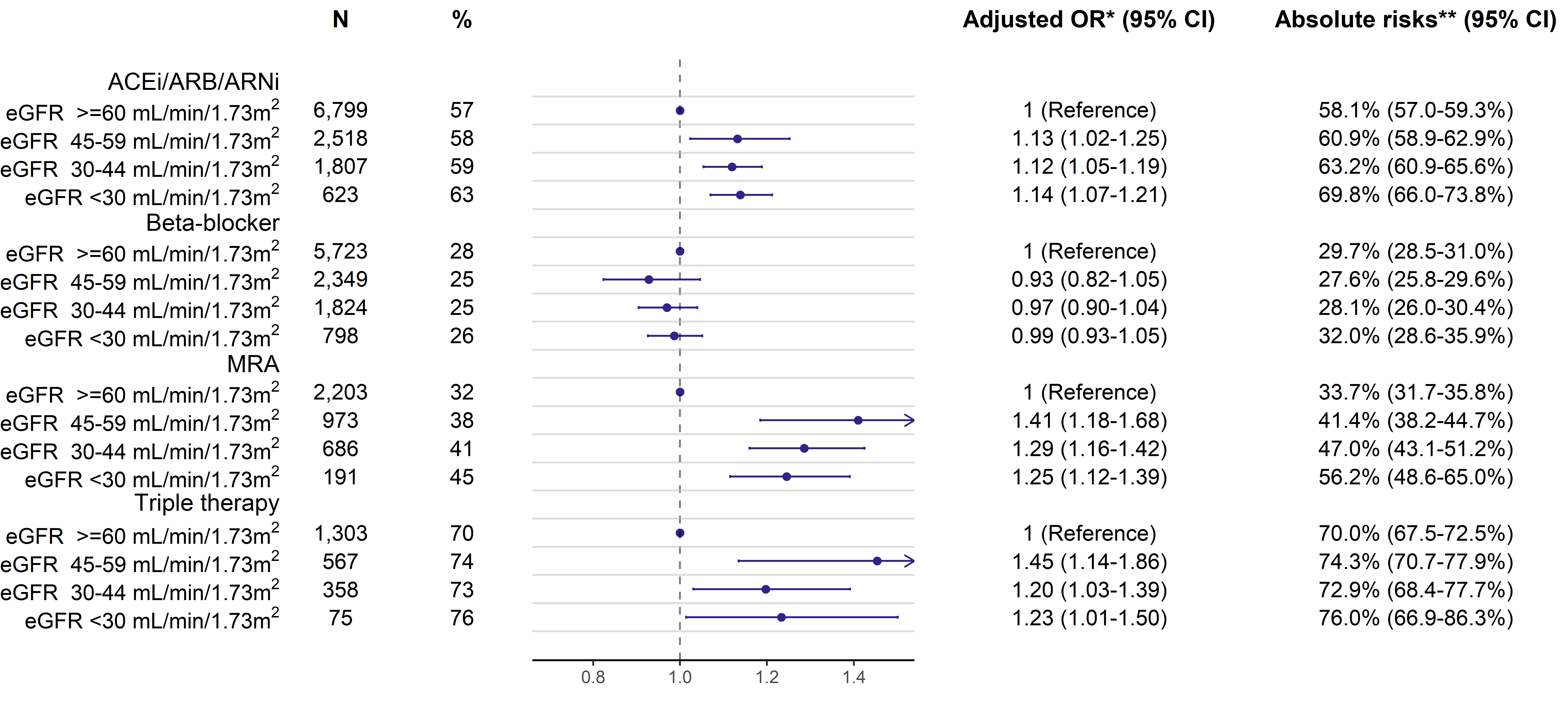


OR, odds ratio; CI, confidence interval; ACEi, angiotensin-converting enzyme inhibitor; ARB, angiotensin receptor blocker; ARNi, angiotensin receptor-neprilysin inhibitor; eGFR, estimated glomerular filtration rate, MRA, mineralocorticoid receptor antagonist.

^*^ Adjusted for age, sex, hospitalization at index, heart failure duration, anemia, atrial fibrillation, cerebrovascular disease, chronic obstructive pulmonary disease, diabetes mellitus, dilated cardiomyopathy, hypertension, ischemic heart disease, liver disease, peripheral artery disease, valvular disease, cancer, coronary revascularization, devices (CRT, ICD, or pacemaker), prescription for digoxin, diuretics, statins, anticoagulants, antiplatelets, or nitrates at index, heart rate, systolic and diastolic blood pressure, hemoglobin, highest achieved education, civil status, income, and year of index category.

^**^ Takes into account censoring and the competing risk of death.

Supplemental figure 5. Multivariable adjusted odds ratios (and 95% confidence intervals) for being prescribed guideline-recommended therapies in patients with heart failure with reduced ejection fraction (HFrEF) and differing eGFR categories in the time periods 2009-2011, 2012-2015, and 2016-2018.


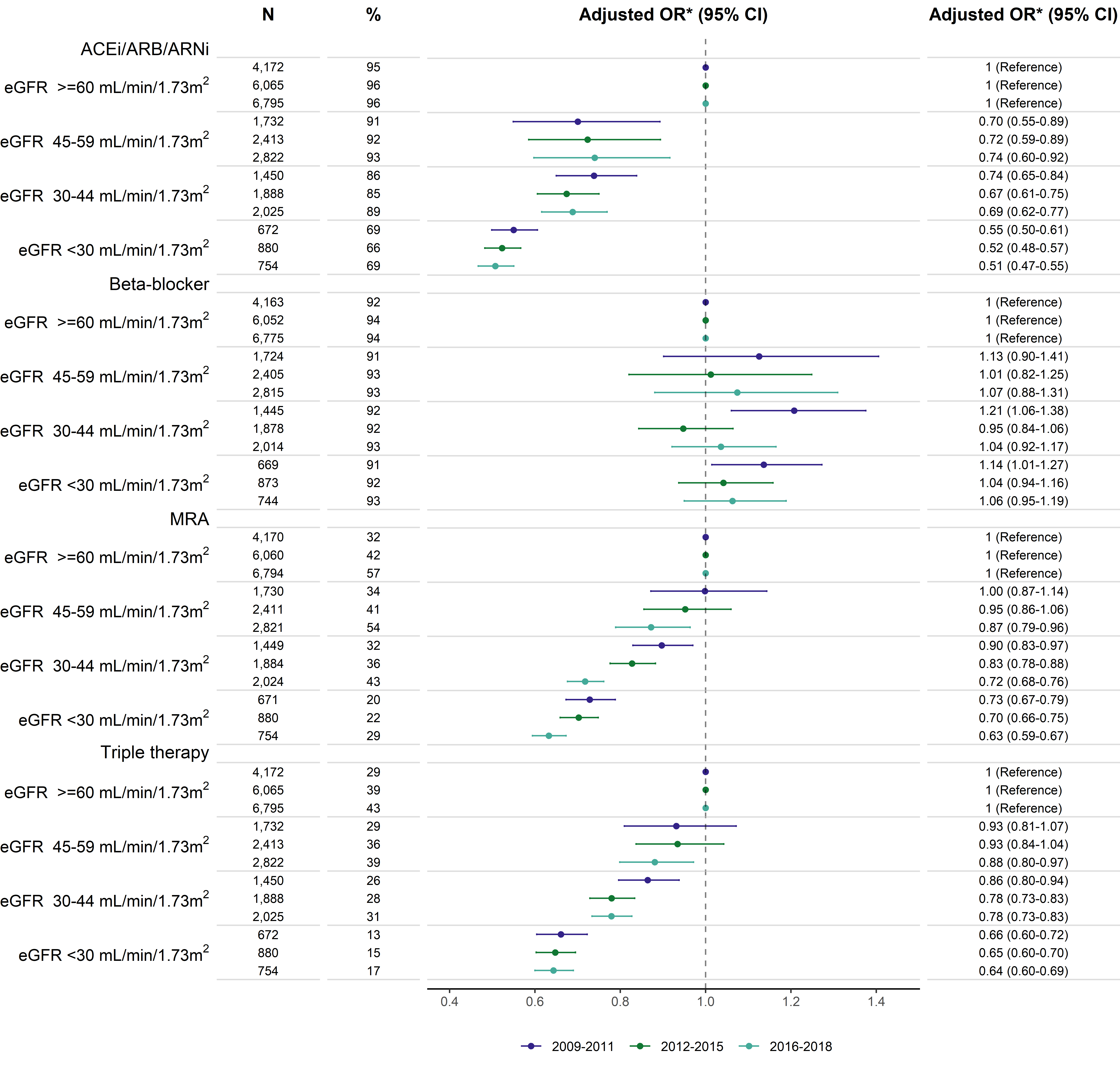


^*^ Adjusted for age, sex, hospitalization at index, heart failure duration, anemia, atrial fibrillation, cerebrovascular disease, chronic obstructive pulmonary disease, diabetes mellitus, dilated cardiomyopathy, hypertension, ischemic heart disease, liver disease, peripheral artery disease, valvular disease, cancer, coronary revascularization, devices (CRT, ICD, or pacemaker), prescription for digoxin, diuretics, statins, anticoagulants, antiplatelets, or nitrates at index, heart rate, systolic and diastolic blood pressure, hemoglobin, highest achieved education, civil status, and income.

Supplemental figure 6. Multivariable adjusted odds ratios (and 95% confidence intervals) for filling prescriptions of guideline-recommended therapies in patients with heart failure with reduced ejection fraction (HFrEF) and differing eGFR categories in the time periods 2009-2011, 2012-2015, and 2016-2018.


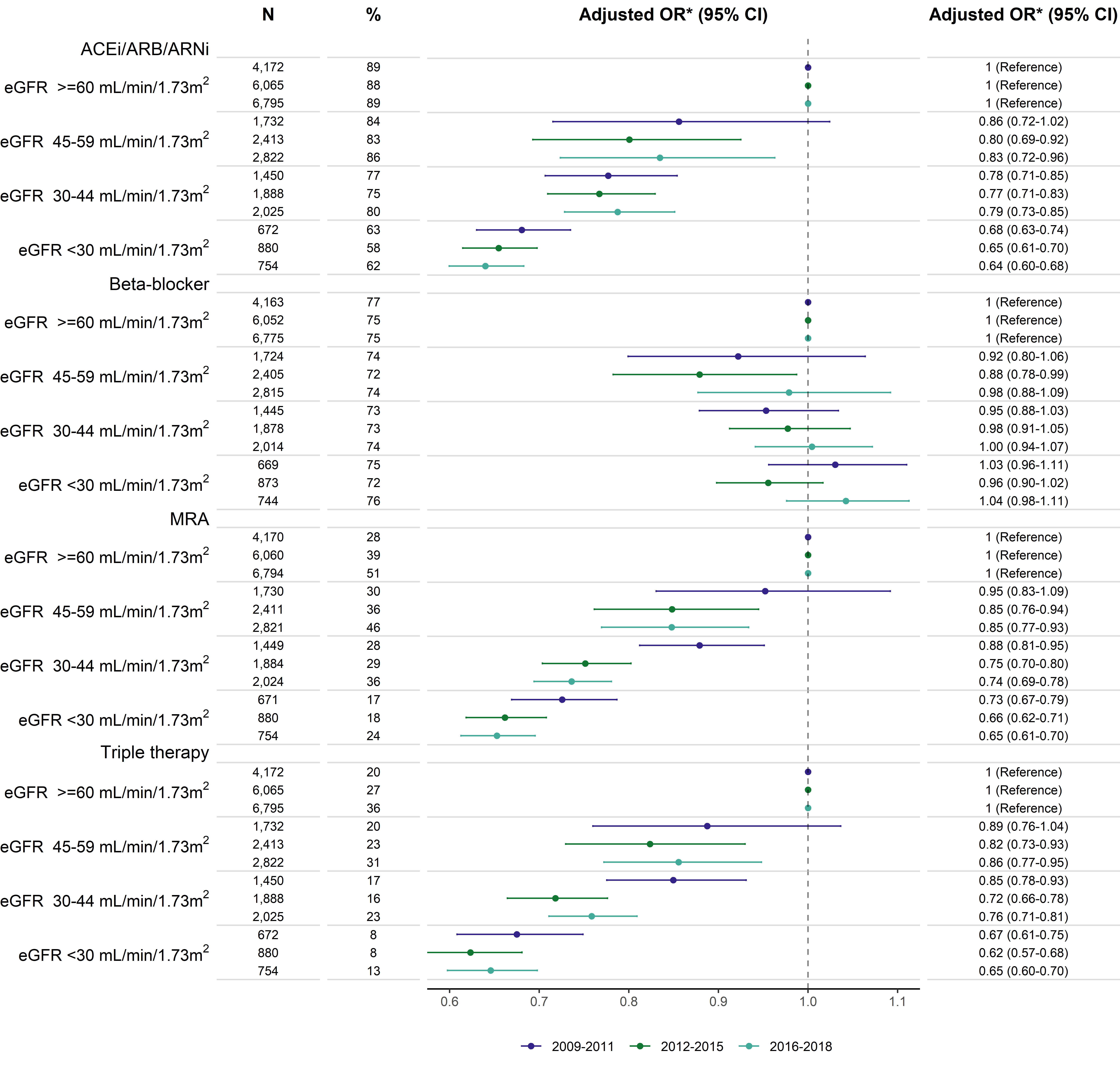


^*^ Adjusted for age, sex, hospitalization at index, heart failure duration, anemia, atrial fibrillation, cerebrovascular disease, chronic obstructive pulmonary disease, diabetes mellitus, dilated cardiomyopathy, hypertension, ischemic heart disease, liver disease, peripheral artery disease, valvular disease, cancer, coronary revascularization, devices (CRT, ICD, or pacemaker), prescription for digoxin, diuretics, statins, anticoagulants, antiplatelets, or nitrates at index, heart rate, systolic and diastolic blood pressure, hemoglobin, highest achieved education, civil status, and income.

Supplemental figure 7A. Multivariable adjusted odds ratios (and 95% confidence intervals) for low adherence (proportion of days covered <80%) to guideline-recommended therapies during the first year of therapy in patients with heart failure with reduced ejection fraction (HFrEF) and differing eGFR categories in the time periods 2009-2011, 2012-2015, and 2016-2018.


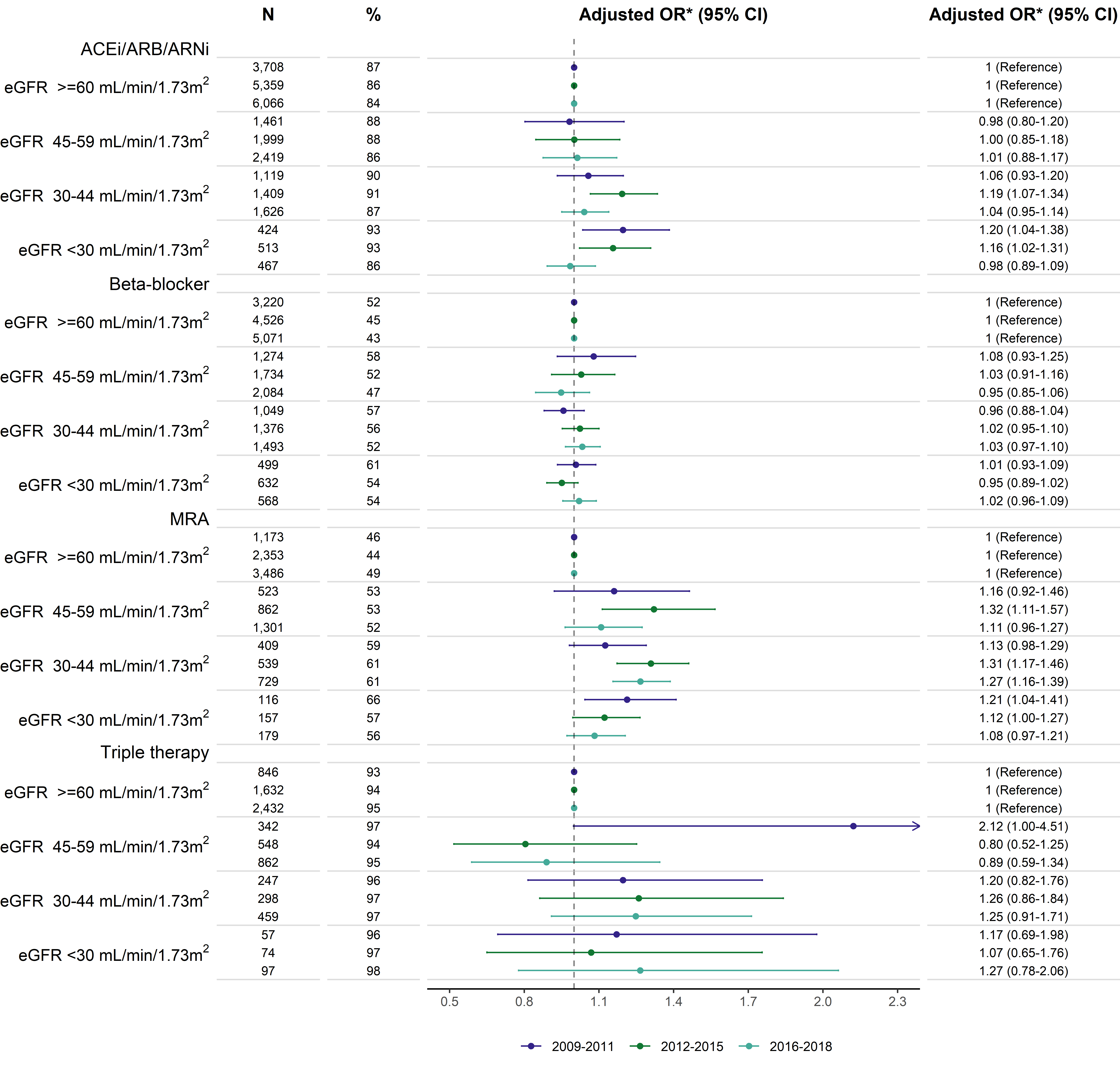


**.**^*^ Adjusted for age, sex, hospitalization at index, heart failure duration, anemia, atrial fibrillation, cerebrovascular disease, chronic obstructive pulmonary disease, diabetes mellitus, dilated cardiomyopathy, hypertension, ischemic heart disease, liver disease, peripheral artery disease, valvular disease, cancer, coronary revascularization, devices (CRT, ICD, or pacemaker), prescription for digoxin, diuretics, statins, anticoagulants, antiplatelets, or nitrates at index, heart rate, systolic and diastolic blood pressure, hemoglobin, highest achieved education, civil status, and income.

Supplemental figure 7B. Multivariable adjusted odds ratios (and 95% confidence intervals) for low persistence (i.e. treatment discontinuation) to guideline-recommended therapies during the first year of therapy in patients with heart failure with reduced ejection fraction (HFrEF) and differing eGFR categories in the time periods 2009-2011, 2012-2015, and 2016-2018.


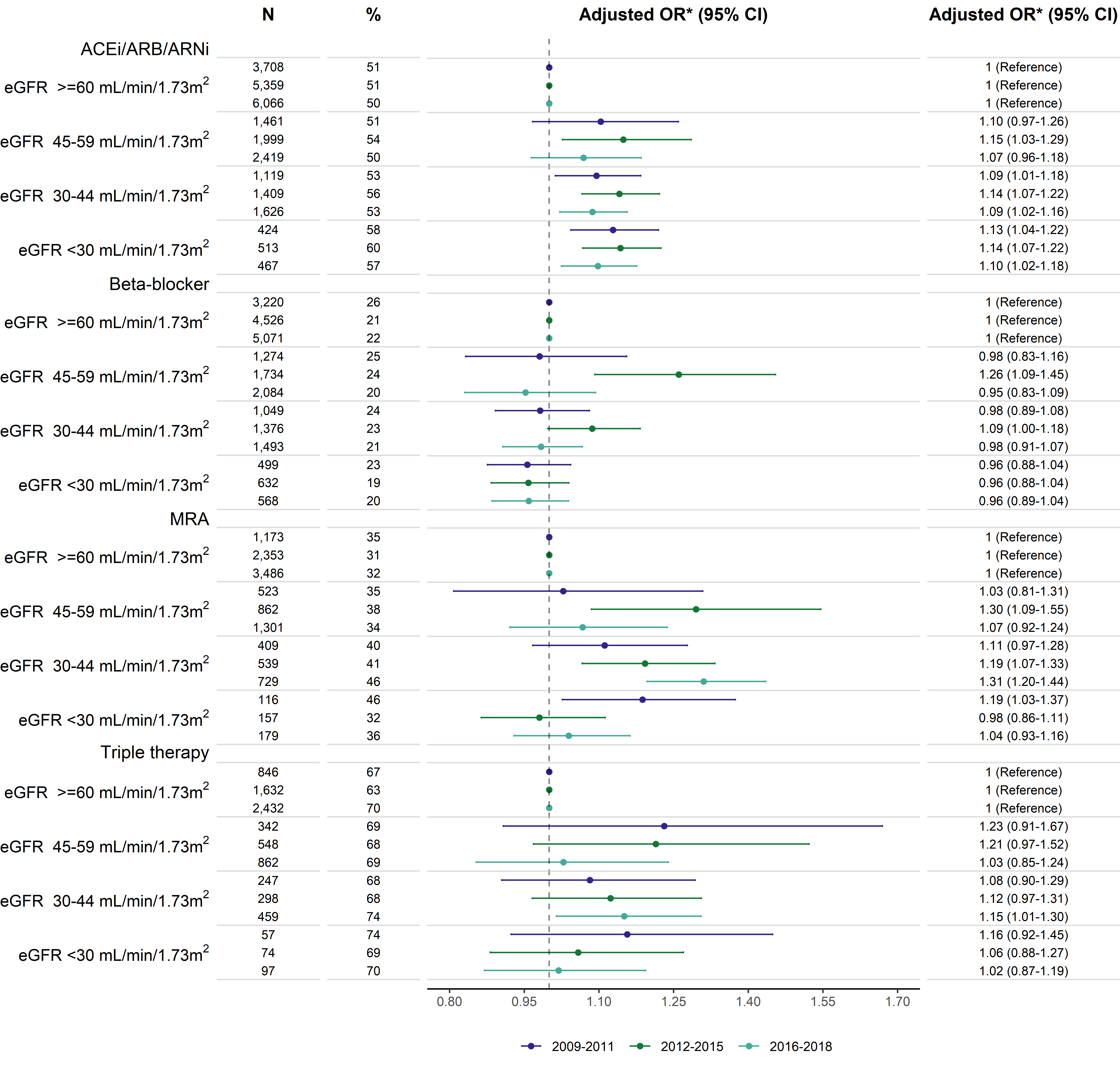


^*^ Adjusted for age, sex, hospitalization at index, heart failure duration, anemia, atrial fibrillation, cerebrovascular disease, chronic obstructive pulmonary disease, diabetes mellitus, dilated cardiomyopathy, hypertension, ischemic heart disease, liver disease, peripheral artery disease, valvular disease, cancer, coronary revascularization, devices (CRT, ICD, or pacemaker), prescription for digoxin, diuretics, statins, anticoagulants, antiplatelets, or nitrates at index, heart rate, systolic and diastolic blood pressure, hemoglobin, highest achieved education, civil status, and income.

Supplemental figure 8. Multivariable adjusted odds ratios (and 95% confidence intervals) for being prescribed guideline-recommended therapies in patients with heart failure with mildly reduced ejection fraction (HFmrEF) and differing eGFR categories in the time periods 2009-2011, 2012-2015, and 2016-2018.


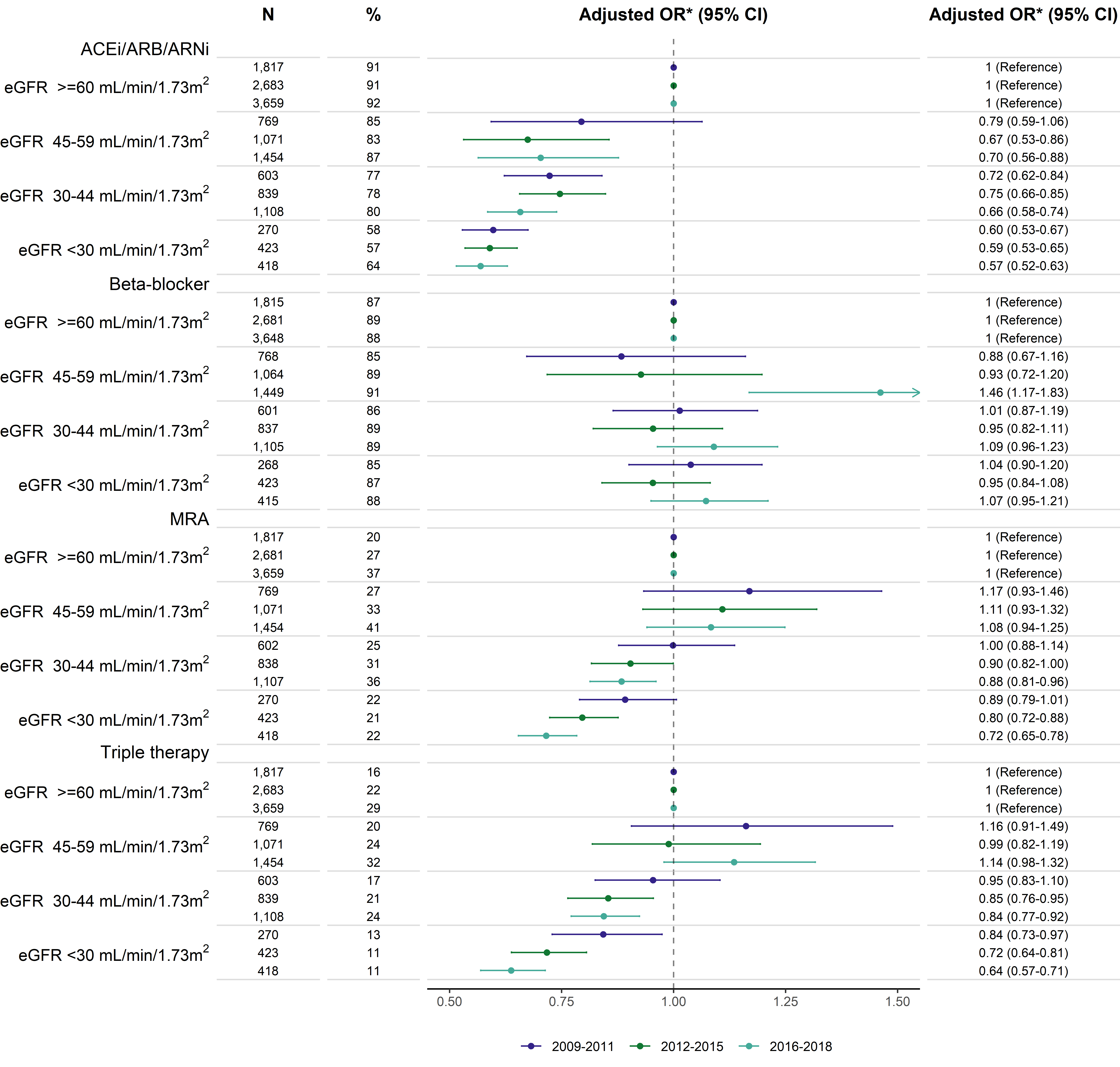


^*^ Adjusted for age, sex, hospitalization at index, heart failure duration, anemia, atrial fibrillation, cerebrovascular disease, chronic obstructive pulmonary disease, diabetes mellitus, dilated cardiomyopathy, hypertension, ischemic heart disease, liver disease, peripheral artery disease, valvular disease, cancer, coronary revascularization, devices (CRT, ICD, or pacemaker), prescription for digoxin, diuretics, statins, anticoagulants, antiplatelets, or nitrates at index, heart rate, systolic and diastolic blood pressure, hemoglobin, highest achieved education, civil status, and income.

Supplemental figure 9. Multivariable adjusted odds ratios (and 95% confidence intervals) for filling prescriptions of guideline-recommended therapies in patients with heart failure with mildly reduced ejection fraction (HFmrEF) and differing eGFR categories in the time periods 2009-2011, 2012-2015, and 2016-2018.


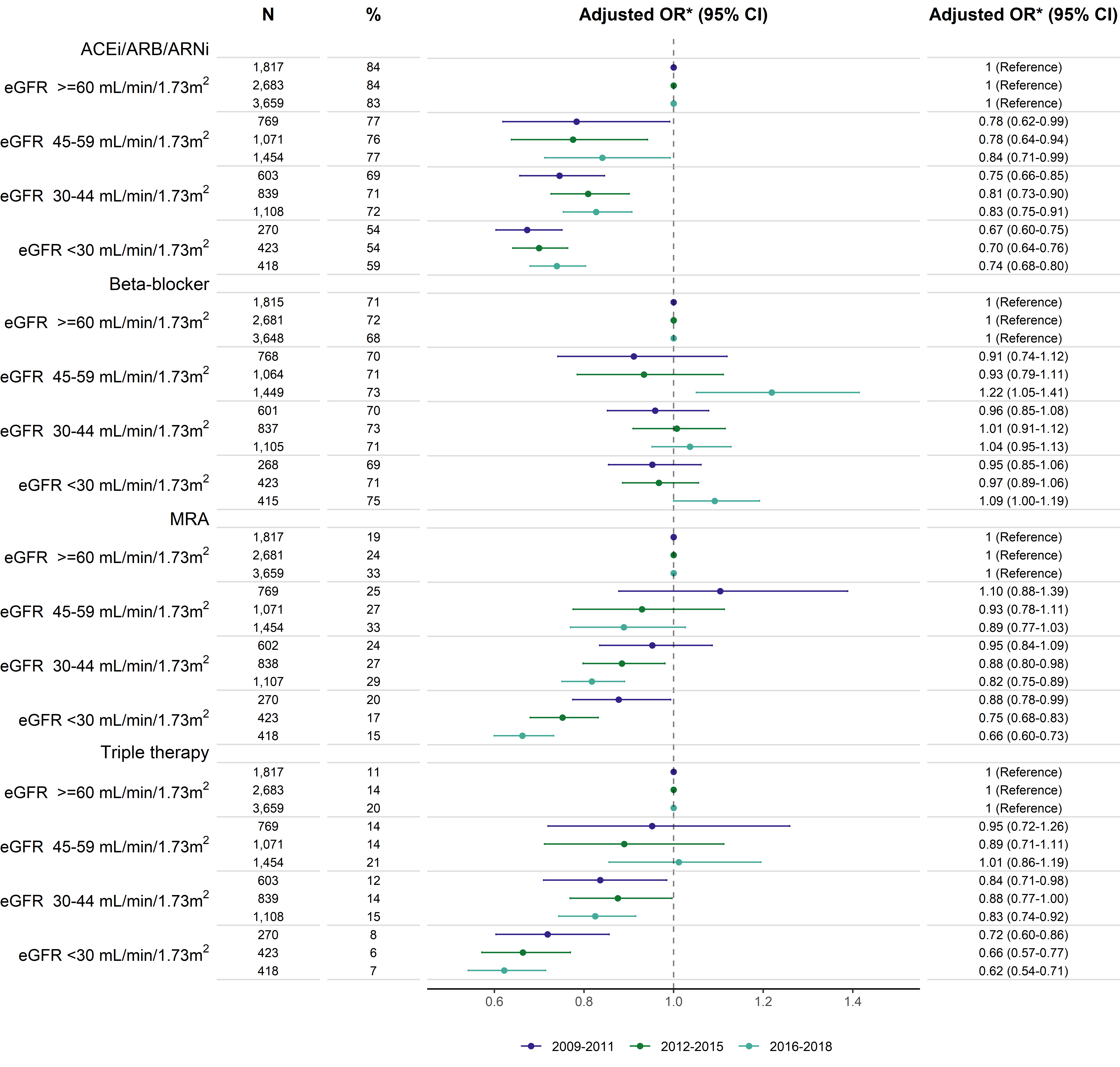


^*^ Adjusted for age, sex, hospitalization at index, heart failure duration, anemia, atrial fibrillation, cerebrovascular disease, chronic obstructive pulmonary disease, diabetes mellitus, dilated cardiomyopathy, hypertension, ischemic heart disease, liver disease, peripheral artery disease, valvular disease, cancer, coronary revascularization, devices (CRT, ICD, or pacemaker), prescription for digoxin, diuretics, statins, anticoagulants, antiplatelets, or nitrates at index, heart rate, systolic and diastolic blood pressure, hemoglobin, highest achieved education, civil status, and income.

Supplemental figure 10A. Multivariable adjusted odds ratios (and 95% confidence intervals) for low adherence (proportion of days covered <80%) to guideline-recommended therapies during the first year of therapy in patients with heart failure with mildly reduced ejection fraction (HFmrEF) and differing eGFR categories in the time periods 2009-2011, 2012-2015, and 2016-2018.

**
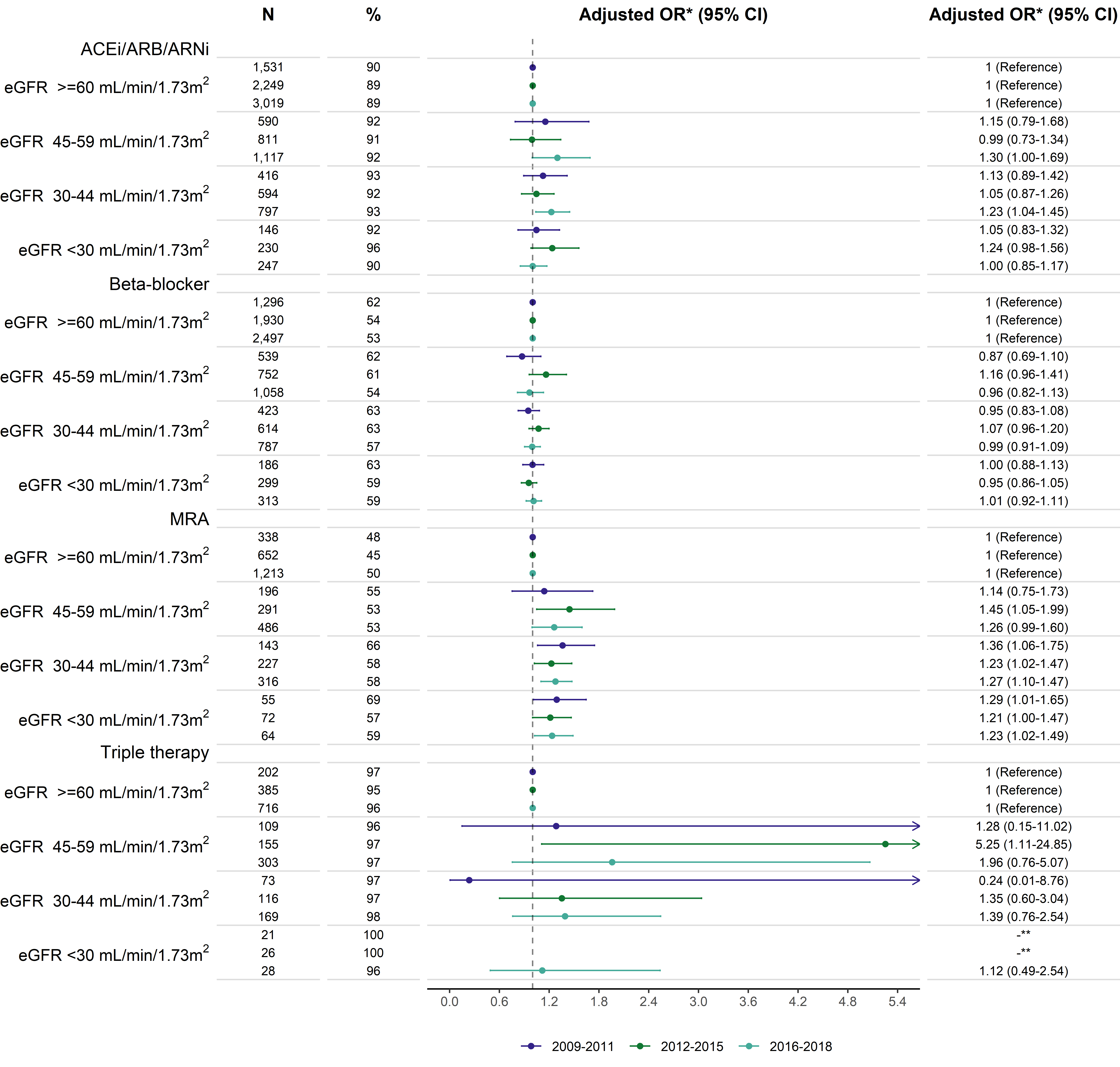
**

^*^ Adjusted for age, sex, hospitalization at index, heart failure duration, anemia, atrial fibrillation, cerebrovascular disease, chronic obstructive pulmonary disease, diabetes mellitus, dilated cardiomyopathy, hypertension, ischemic heart disease, liver disease, peripheral artery disease, valvular disease, cancer, coronary revascularization, devices (CRT, ICD, or pacemaker), prescription for digoxin, diuretics, statins, anticoagulants, antiplatelets, or nitrates at index, heart rate, systolic and diastolic blood pressure, hemoglobin, highest achieved education, civil status, and income.

^**^ Sample did not allow the fitting of a valid model.

Supplemental figure 10B. Multivariable adjusted odds ratios (and 95% confidence intervals) for low persistence (i.e. treatment discontinuation) to guideline-recommended therapies during the first year of therapy in patients with heart failure with mildly reduced ejection fraction (HFmrEF) and differing eGFR categories in the time periods 2009-2011, 2012-2015, and 2016-2018.


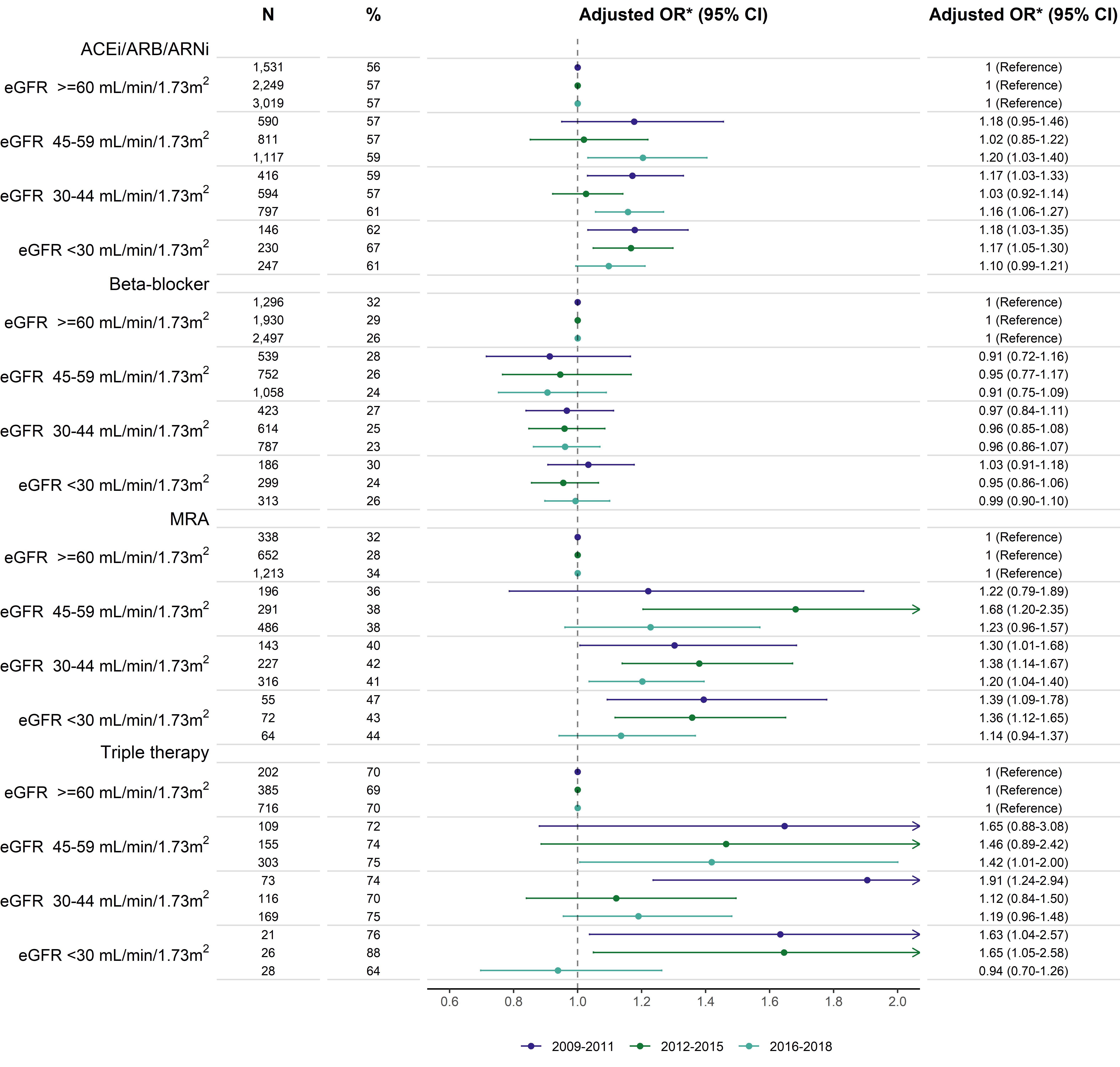


^*^ Adjusted for age, sex, hospitalization at index, heart failure duration, anemia, atrial fibrillation, cerebrovascular disease, chronic obstructive pulmonary disease, diabetes mellitus, dilated cardiomyopathy, hypertension, ischemic heart disease, liver disease, peripheral artery disease, valvular disease, cancer, coronary revascularization, devices (CRT, ICD, or pacemaker), prescription for digoxin, diuretics, statins, anticoagulants, antiplatelets, or nitrates at index, heart rate, systolic and diastolic blood pressure, hemoglobin, highest achieved education, civil status, and income.
